# Maternal Parenting Electronic Diary in the Context of a Home Visit Intervention for Adolescent Mothers in an Urban Deprived Area of São Paulo, Brazil: Randomized Controlled Trial

**DOI:** 10.2196/13686

**Published:** 2020-07-28

**Authors:** Daniel Fatori, Adriana Argeu, Helena Brentani, Anna Chiesa, Lislaine Fracolli, Alicia Matijasevich, Euripedes C Miguel, Guilherme Polanczyk

**Affiliations:** 1 Department of Psychiatry University of Sao Paulo Medical School Sao Paulo Brazil; 2 Collective Health Nursing Department School of Nursing University of Sao Paulo Sao Paulo Brazil; 3 Department of Preventive Medicine University of Sao Paulo Medical School Sao Paulo Brazil

**Keywords:** mHealth, early childhood development, maternal care, randomized clinical trial, daily diary, ambulatory assessment

## Abstract

**Background:**

Pregnancy during adolescence is prevalent in low- and middle-income countries (LMICs), which is associated with various adverse outcomes that can be prevented with home visiting programs. However, testing these interventions in LMICs can be challenging due to limited resources. The use of electronic data collection via smartphones can be an alternative and ideal low-cost method to measure outcomes in an environment with adverse conditions.

**Objective:**

Our study had two objectives: to test the efficacy of a nurse home visiting intervention on maternal parenting and well-being measured by an electronic daily diary (eDiary), and to investigate the compliance rate of the eDiary measurement method.

**Methods:**

We conducted a randomized controlled trial to test the efficacy of Primeiros Laços, a nurse home visiting program, for adolescent mothers living in an urban deprived area of São Paulo, Brazil. A total of 169 pregnant adolescents were assessed for eligibility criteria, 80 of whom were included and randomized to the intervention (n=40) and control group (care as usual, n=40). Primeiros Laços is a home visiting intervention delivered by trained nurses tailored to first-time pregnant adolescents and their children, starting during the first 16 weeks of pregnancy until the child reaches 24 months of age. Participants were assessed by blind interviewers at 8-16 weeks of pregnancy (baseline), 30 weeks of pregnancy, and when the child was 3, 6, and 12 months of age. At 18 months, participants were assessed regarding maternal parenting and parental well-being using a 7-consecutive-day eDiary. The smartphone app was programmed to notify participants every day at 9:00 PM over a period of seven days.

**Results:**

We were able to contact 57/80 (71%) participants (29 from the intervention group and 28 from the control group) when the child was 18 months of age. Forty-eight of the 57 participants (84%) completed at least one day of the eDiary protocol. The daily compliance rate ranged from 49% to 70%. Our analyses showed a significant effect of the intervention on parental well-being (B=0.32, 95% CI [0.06, 0.58], *P*=.02) and the maternal parenting behavior of the mother telling a story or singing to the child (odds ratio=2.33, 95% CI [1.20, 4.50], *P*=.01).Our analyses showed a significant effect of the intervention on parental well-being (B=0.32, *P*=.02) and the maternal parenting behavior of the mother telling a story or singing to the child (odds ratio=2.33, *P*=.01).

**Conclusions:**

The Primeiros Laços intervention improved maternal parenting and parental well-being, demonstrating its promise for low-income adolescent mothers. The compliance rate of the eDiary assessment showed that it was generally accepted by adolescent mothers with limited resources. Future studies can implement ambulatory assessment in LMICs via smartphones to measure mother and child behaviors.

**Trial Registration:**

ClinicalTrials.gov NCT02807818; https://clinicaltrials.gov/ct2/show/NCT02807818

## Introduction

Every year, 16 million 15 to 19-year-old adolescents give birth, predominantly in low and middle-income countries (LMICs) [1]. Pregnancy during adolescence is associated with higher risks of eclampsia, endometritis, infections, low birth weight [2], increased maternal and neonatal mortality [3], and morbidity such as maternal depression, parental stress, and insecure attachment [4,5]. Children of adolescent mothers are also at increased risk for impaired early childhood development and behavioral functioning, as well as insecure and disorganized attachment. Interaction between adolescent mothers and their children is reported to be different from that of adult mothers. In particular, adolescent mothers have been shown to provide less verbal stimulation and are less sensitive to the child’s needs [6]. Hence, developing and testing interventions focused on preventing adverse outcomes among this high-risk group is a timely need, especially in LMICs where an estimated 250 million children below the age of 5 do not meet their potential [7]. The basis of robust intervention studies is the adequate and accurate measurement of outcomes. However, testing early childhood development (ECD) interventions in LMICs can be challenging due to limited resources.

Studies focusing on maternal parenting usually rely on direct or recorded observations of dyads by trained and certified experts [8] or self-reported questionnaires based on retrospective behavior. However, these methods have important limitations. Observation of dyads is a lengthy and costly assessment, which is difficult to implement in contexts where resources (eg, trained experts, available space) are scarce and accessibility is limited, such as in LMICs. Retrospective self-reported questionnaires are subject to memory bias and lack ecological validity, resulting in potentially inaccurate data (information bias). The use of ambulatory assessment delivered via smartphones can be an alternative data collection method to circumvent these problems.

Ambulatory assessment encompasses methods designed to study people in their natural environment. Owing to the ubiquitous presence of smartphones, ambulatory assessment methods are now most commonly delivered via mobile technology. Subjects can be notified to answer questions during specific times of the day throughout days, weeks, or months. Given the numerous advantages of ambulatory assessment, its use has recently grown in many fields [9-11]. Nevertheless, the field of ECD has been slow in adopting ambulatory assessment, with few initiatives conducted in developed countries. One study used the ambulatory assessment method to assess the relationship between crying and fussing at 12 months of age along with the physical health of the child and emotional security of the mother [12]. Another study validated an ambulatory assessment protocol to evaluate parental discipline in children aged 18-36 months [13]. However, to our knowledge, no study conducted in an LMIC has tested the potential of ambulatory assessment in the context of ECD programs. Moreover, ambulatory assessment methods have only been adopted in observational studies to date, and there has been no randomized trial in the ECD field that used ambulatory assessment via mobile technology as an outcome. Most studies testing mobile technology in the field of ECD are interventions related to various outcomes such as maternal depression [14], infant feeding [15-17], physical activity [18], and maternal health [19-22].

Therefore, we implemented an electronic daily diary (eDiary), a specific type of ambulatory assessment through which participants report a set of behaviors that occurred during the day to measure maternal parenting and parental well-being, in the context of a randomized controlled trial. In particular, we assessed the efficacy of Primeiros Laços, a nurse home visiting program for adolescent mothers living in an urban deprived area of São Paulo, Brazil. The intervention aimed to foster the mother-child relationship and improve child development. The present study had two objectives: (1) to test the efficacy of a nurse home visiting intervention on child maternal parenting and parental well-being measured by an eDiary, and (2) to investigate the compliance rate of the eDiary measurement method. The first hypothesis was that mothers who receive the home visiting intervention will present a higher frequency of maternal parenting behaviors and higher scores of well-being. The second hypothesis was that the eDiary method will present a compliance rate similar to that reported in previous studies, despite the fact that our sample is composed of low-income adolescent mothers living in adverse conditions.

## Methods

### Study Design and Participants

The present study is part of a parallel group randomized controlled trial that was originally designed to test the efficacy of Primeiros Laços, a nurse home visiting program for adolescent mothers living in an urban deprived area of São Paulo, Brazil. Previous findings from this trial can be found elsewhere [23-26]. From June to September 2015, a total of 169 pregnant youth were assessed for eligibility criteria, 80 of whom were included and randomized to the intervention (n=40) and control (care as usual, n=40) groups. Inclusion criteria were: (a) aged 14 to 19 years old, (b) first pregnancy, (c) between 8-16 weeks of gestation, (d) low socioeconomic status (classes C, D, E according to the widely used Brazilian ABEP scale [27]), and (e) living in the western region of the city of São Paulo. Participants were recruited in the primary health care system. To avoid unbalanced groups, randomization was stratified according to the primary health care unit type and grandmother years of schooling. The allocation ratio was 1:1.

### Intervention

Primeiros Laços is a home visiting intervention delivered by trained nurses that is tailored to first-time adolescent pregnant women and their children, which starts during the first 16 weeks of pregnancy until the child reaches 24 months of age. The frequency of visits was (a) biweekly during gestation and from 2 to 20 months of the child’s age; (b) weekly during the first and last month of pregnancy, and during puerperium; and (c) monthly from 21 to 24 months of the child’s age. The Primeiros Laços program is based on three theoretical frameworks: attachment theory [28], self-efficacy theory [29], and the bioecological model [30]. This intervention adopts a learning-based approach and aims to have the child at the center of the mother’s life, fostering her capacity to perceive and react to the child’s needs, with the objective of improving maternal sensitivity to the child’s behaviors and emotions. Primeiros Laços was developed by our team based on the Brazilian program Janelas de Oportunidades [31,32], Minding the Baby program [33], and Nurse-Family Partnership [34]. The development of the program was also informed with input from key national stakeholders involved in early childhood and maternal health research and advocacy.

The content is directed to five domains. The first domain is health and social care, in which the nurse provides education regarding maternal and child health such as nutrition, hygiene, common pathologies in childhood, domestic care, vaccination, prevention of accidents, and child development. The second domain is environmental health, in which the nurse provides support and help to identify resources to guarantee adequate living conditions, safe housing, daycare and school, and access to health services. The third domain is life course, which involves life course planning to help participants achieve life goals such as finishing high school, finding a part-time job, starting college, and postponing the birth of a second child. The needs and goals of the participants were discussed individually to respect their personal goals and wishes. Nurses also help mothers gain access to primary care services and government-sponsored social programs. The fourth domain includes parenting skills, which involves education on parenting skills and child behavior considering each development stage, aiming to develop a sensitive and responsive pattern of care. The last domain is family and social support, as nurses frequently highlighted the role of family members and friends to support parental needs. Nurses were supervised weekly by the developers of the program (senior nurses and a child psychologist) to discuss cases, plan visits, and to guarantee fidelity and quality of visits. More details about the intervention can be found elsewhere [35,36].

### Care as Usual

Participants allocated to the control group received care from Unified Health System (Sistema Único de Saúde), Brazil’s public health system [37], according to national guidelines of the Ministry of Health [38-40] in line with World Health Organization guidelines. Prenatal and postnatal care is delivered by health units of the primary care system free of charge, focusing on preventive interventions, early detection of gestational risk, and referral to specialized health services in cases of high-risk pregnancies. Participants from the intervention group also had access to public health care provided by the Unified Health System.

### Assessment

Participants were assessed by interviewers blinded to group allocation at 8-16 weeks of pregnancy (baseline), 30 weeks of pregnancy, and when the child was 3, 6, 12, 18, and 24 months of age. Interviews were conducted by trained psychologists who underwent a 1-month training program provided by senior psychiatrists, psychologists, and pediatricians. Before study interviews commenced, interviewers trained by assessing volunteers living in the western region of São Paulo. Ambulatory assessment was administered when the child was 18 months old. At baseline (8-16 weeks of pregnancy), we measured depression symptoms using the Beck Depression Inventory [41,42] and anxiety symptoms were assessed using the Beck Anxiety Inventory [43,44], which have both been validated in Brazil. Family food insecurity was measured using the abridged version of the Brazilian Food Insecurity Scale (Escala de Insegurança Alimentar) [45], a widely used scale adopted in national epidemiological studies [46].

### eDiary

We used the LifeData system [47], a mobile technology suite for smartphone secure data collection available for Android and iOS. Our eDiary protocol comprised a set of questions designed via a web-based dashboard. The smartphone app was programmed to notify participants every day at 9:00 PM over a period of 7 days. Participants had up to 120 minutes to complete the eDiary protocol and received up to three reminders. Trained psychologists visited participants to provide support on downloading, installing, and using the app. The eDiary protocol was shown and explained to all participants using a demonstration version installed on the psychologist’s smartphone. Participants who did not own a smartphone were provided one for the duration of the eDiary protocol (n=18).

The present study focused on the following questions of the eDiary protocol: (1) How do you feel about the day today? (score 1-100, general maternal well-being) (2) Did you take care of your child or spend some time with him/her today? (3) What was it like taking care of your child today? (score 1-100, parental well-being) (4) Did you read or show a book to your child today? (5) Did you tell stories or sing to your child today? (6) Did you go out or go for a stroll with your child today? (7) Did you play with your child today? (8) Did you talk to your child today? (9) Did you eat or have a meal with your child today? (10) Did you kiss, hug, tickle (or have any other physical contact with) your child today? (11) How many hours did you spend with your child today? Maternal parenting behavior was operationalized as parenting practices related to mother-child interaction and positive activities known to stimulate child development. Questions 2, and 4 to 11 were related to maternal parenting. Screenshots of the eDiary protocol can be found elsewhere (imgur.com/a/eTMAp84).

### Statistical Analysis

The sample size was calculated based on the difference in the electroencephalogram alpha wave frequency between the groups (30%) with a probability of type I error of 5% and statistical power of 80%. Frequencies and distribution of eDiary outcomes are provided for each of the 7 days of the intervention. The Fisher exact test and *t* tests were used to examine differences between groups (intervention vs control) at each time point. To examine intervention effects on eDiary outcomes, we used generalized estimating equation models [48]. Continuous outcomes were normalized using z-scores. Generalized estimating equation models were used to examine mean differences in normalized continuous outcomes and differences in predictive probabilities for categorical outcomes, both over multiple time points. The quasi-likelihood under the independence model criterion was used to indicate adequate correlation structures for longitudinal data that show a better fit to each model [49]. Fitted models were used to estimate and plot marginal mean scores for continuous outcomes and predictive probabilities for categorical outcomes at each time point. Time was entered as a continuous covariate in all models. Time trends were verified, and all models presented a linear trend [50]. All randomized participants that were contacted when their child was 18 months of age were included in the analysis, except for participants with missing data at all time points (n=9). Statistical tests were all two-sided and *P* values <.05 were considered statistically significant. Parameter estimates are reported with the 95% CI. Analyses were conducted using STATA 15.1 software.

### Ethical Considerations

Our study was approved by the Ethics Committee of the University of São Paulo Medical School, University Hospital of the University of São Paulo, and São Paulo Municipal Health Department. All participants and their primary caregiver signed written informed consent forms. The study was registered at clinicaltrials.gov (NCT02807818).

## Results

**Figure 1:**
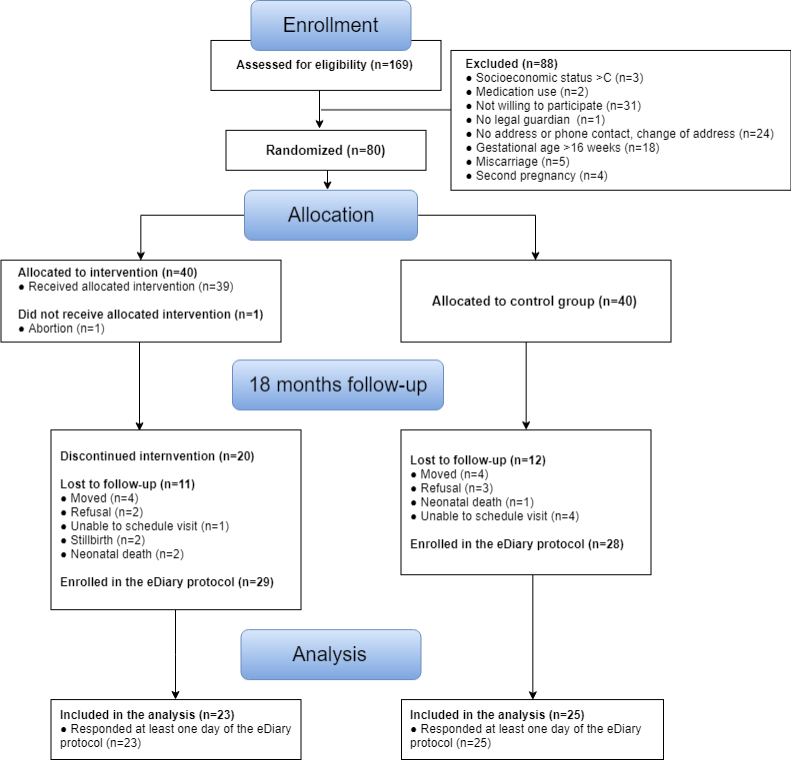
Consolidated Standards of Reporting Trials (CONSORT) diagram.

**Figure 2:**
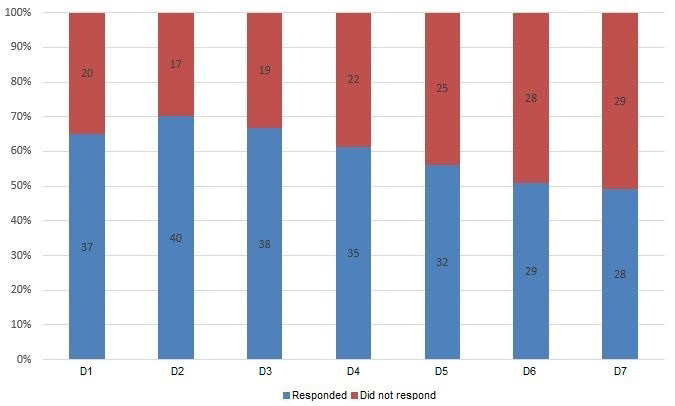
eDiary compliance rate per day (n=57).

We were able to contact 57/80 (71%) participants (29 in the intervention group and 28 in the control group) when the child was 18 months old. Figure 1 shows the Consolidated Standards of Reporting Trials (CONSORT) flow chart for participant selection and grouping throughout the trial. Twenty participants discontinued the intervention. Forty-eight of the 57 (84%) participants completed at least one day of the eDiary protocol. The daily compliance rate ranged from 49% to 70% (Figure 2). We tested for potential baseline differences between the analysis group (n=48) and the remaining participants (n=32) but did not find statistically significant differences (Table 1). In addition, there was no association of specific days of the week with response status. Participants who received a smartphone for the duration of the study (n=18) also did not show a different response rate from those who already owned a smartphone.

**Table 1:** Baseline sample characteristics (N=80).

Baseline characteristics	Included for analysis (n=48)	Lost to follow up (n=32)	*P* value
Intervention group, n (%)	23 (48)	17 (53)	.82
Maternal age, mean (SD)	17.0 (1.4)	17.2 (0.9)	.54
Family SES^a^ (D and E status), n (%)	16 (33)	14 (44)	.36
Maternal educational level (illiterate), n (%)	5 (10)	7 (22)	.21
Grandmother educational level (illiterate), n (%)	24 (10)	19 (44)	.49
Family income (0-300), n (%)	2 (4)	1 (4)	.85
Substance use during gestation, n (%)	17 (35.)	10 (31)	.81
Food insecurity, n (%)	21 (44)	13 (41)	.82
Maternal depression, n (%)	12 (25)	5 (16)	.41
Maternal anxiety, n (%)	13 (27)	6 (19)	.43
Child sex (male), n (%)	25 (52)	15 (56)	.81

^a^SES: socioeconomic status.

**Figure 3:**
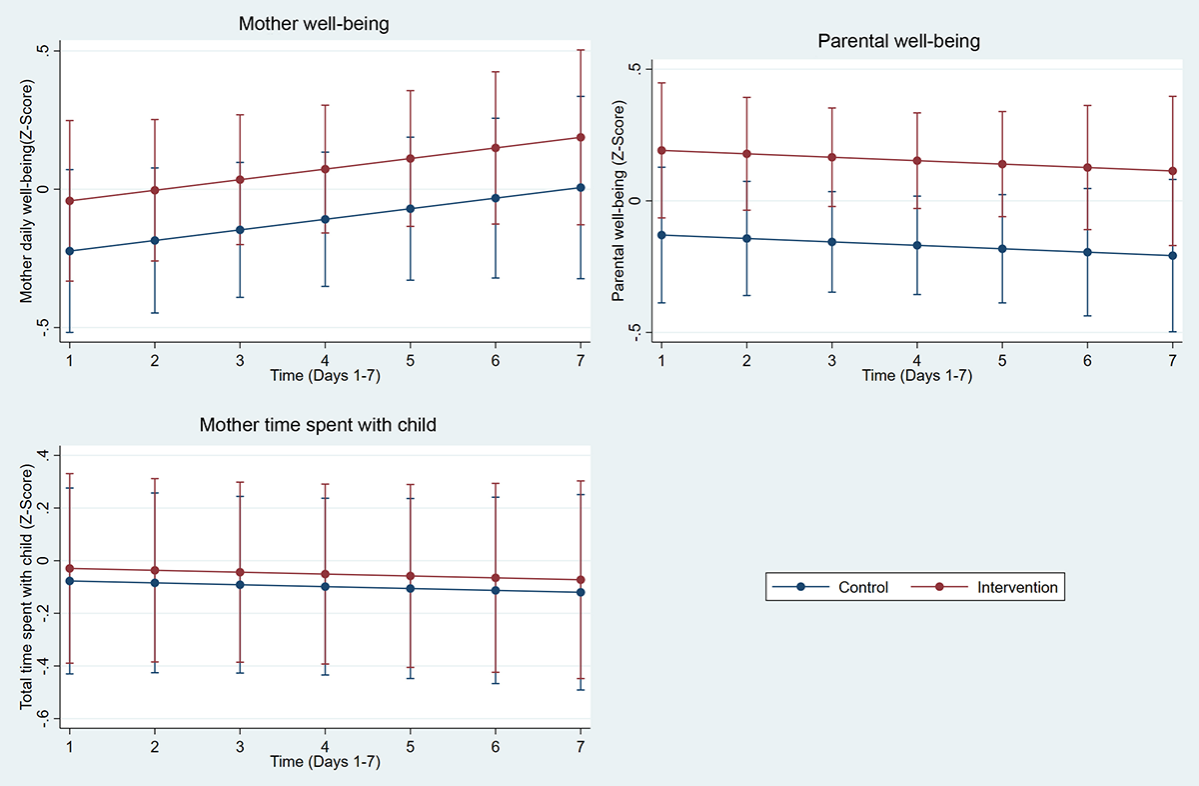
Effect of intervention on continuous outcomes: mother well-being (B=0.18, 95% CI [-0.15, 0.51],*P*=.286), parental well-being (B=0.32, 95% CI [0.06, 0.58], *P*=.015), total time spent with the child (B=0.05, 95% CI [-0.43, 0.52], *P*=.845).

**Figure 4:**
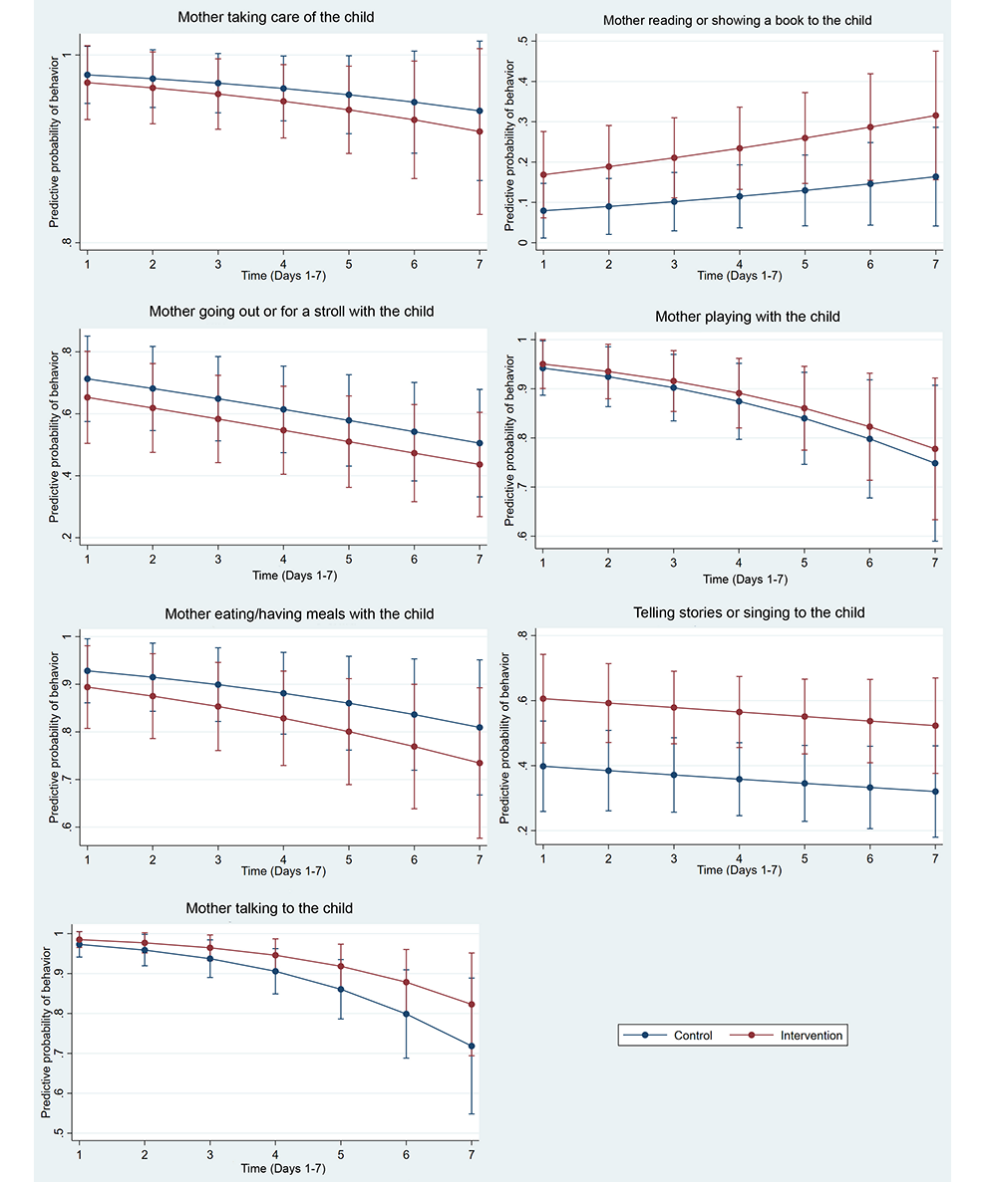
Effect of intervention on categorical outcomes: mother taking care of the child (OR=0.71, 95% CI [0.19, 2.60], *P*=.61), mother reading or showing a book to the child (OR=2.35, 95% CI [0.90, 6.11], *P*=.08), mother telling stories or singing to the child (OR=2.33, 95% CI [1.20, 4.50]. *P*=.01), mother going out or for a stroll with the child (OR=0.76, 95% CI [0.33, 1.72], *P*=.51), mother playing with the child (OR=1.17, 95% CI [0.44, 3.17], *P*=.75), mother talking to the child (OR=1.82, 95% CI [0.69. 4.77], *P*=.22), mother eating/having meals with the child (OR=0.65, 95% CI [0.22, 1.90], *P*=.43).

Tables 2 and 3 depict the total sample distribution of eDiary data, as well as the distribution for each group. The total sample grand mean for continuous outcomes across the 1-week period was 80.3 for general well-being, 89.1 for parental well-being, and 843.8 for time spent in minutes with the child. Univariate analysis showed the following significant associations: time spent with the child at day 4 (*P*=.02), read to child at day 6 (*P*=.04), and telling a story or singing at day 2 (*P*=.04) and at day 6 (*P*=.047). Figure 3 and Figure 4 depict the results of the generalized estimating equations models with trajectories of outcomes. Overall, the results showed a significant effect of the intervention on parental well-being (B=0.32, 95% CI [0.06, 0.58], *P*=.01) and increased behavior of the mother telling a story or singing to the child (odds ratio=2.33, 95% CI [1.20, 4.50], *P*=.01) (Figure 4).

**Table 2:** Results of continuous outcomes per day.

Outcome		Day 1		Day 2		Day 3		Day 4		Day 5		Day 6		Day 7	
**General well-being, mean (SD)**															
	TS^a^		80.1 (22.7)		84.0 (19.0)		72.1 (28.1)		74.1 (24.6)		86.1 (21.4)		84.7 (17.9)		81.3 (27.6)
	C^b^		79.3 (20.9)		83.8 (16.7)		68.9 (27.7)		72.4 (24.8)		87.3 (15.3)		79.7 (17.2)		77.3 (24.5)
	I^c^		80.8 (24.8)		84.0 (21.2)		74.9 (28.9)		76.3 (25.2)		84.9 (26.9)		89.1 (17.9)		84.3 (30.2)
**Parental well-being, mean (SD)**															
	TS		90.0 (14.0)		93.9 (10.7)		85.1 (20.5)		83.0 (20.3)		90.7 (12.1)		93.4 (12.6)		87.4 (18.8)
	C		88.7 (13.8)		91.0 (13.1)		82.8 (22.1)		81.3 (20.8)		88.7 (13.6)		86.9 (16.1)		84.6 (15.6)
	I		91.1 (14.4)		96.6 (7.1)		87.3 (19.1)		85.0 (20.2)		92.9 (10.3)		99.3 (1.5)		89.5 (21.1)
**Time spent with child (minutes), mean (SD)**															
	TS		868.7 (500.3)		819.2 (519.8)		676.2 (572.1)		978.5 (482.4)		921.0 (500.9)		867.4 (533.0)		775.5 (588.5)
	C		851.1 (458.4)		817.5 (496.3)		691.0 (554.3)		804.4 (493.5)		955.7 (450.1)		712.4 (489.0)		821.8 (525.2)
	I		884.4 (547.2)		820.7 (554.7)		663.0 (602.3)		1189.9 (386.6)		883.9 (563.6)		1010.5 (550.2)		742.5 (647.3)

^a^TS: total sample.

^b^C: control group.

^c^I: intervention group.

**Table 3:** Frequency of categorical outcomes per day.a

Outcome		Day 1	Day 2	Day 3	Day 4	Day 5	Day 6	Day 7
**Taking care of the child, n (%)**								
	TS^b^	36 (97)	37 (95)	37 (97)	32 (97)	31 (100)	25 (89)	24 (92)
	C^c^	17 (47)	18 (49)	18 (49)	17 (53)	16 (52)	12 (48)	10 (42)
	I^d^	19 (53)	19 (51)	19 (51)	15 (47)	15 (48)	13 (52)	14 (58)
**Reading or showing a book to the child, n (%)**								
	TS	4 (11)	6 (16)	5 (14)	7 (22)	8 (26)	5 (20)	6 (25)
	C	1 (25)	2 (33)	2 (40)	3 (43)	3 (38)	0	2 (33)
	I	3 (75)	4 (67)	3 (60)	4 (57)	5 (63)	5 (100)	4 (67)
**Telling stories or singing to the child, n (%)**								
	TS	19 (53)	23 (62)	18 (49)	12 (38)	14 (45)	12 (48)	11 (46)
	C	7 (37)	8 (35)	8 (44)	6 (50)	6 (43)	3 (25)	4 (36)
	I	12 (63)	15 (65)	10 (56)	6 (50)	8 (57)	9 (75)	7 (64)
**Going out or for a stroll with the child, n (%)**								
	TS	29 (81)	18 (49)	23 (62)	20 (63)	23 (74)	12 (48)	12 (50)
	C	16 (55)	8 (44)	14 (61)	12 (60)	12 (52)	4 (33)	3 (25)
	I	13 (45)	10 (56)	9 (39)	8 (40)	11 (48)	8 (67)	9 (75)
**Playing with the child, n (%)**								
	TS	36 (100)	33 (89)	35 (46)	31 (97)	29 (94)	20 (80)	20 (83)
	C	17 (47)	15 (45)	17 (49)	16 (52)	15 (52)	9 (45)	8 (40)
	I	19 (53)	18 (53)	18 (51)	15 (48)	14 (48)	11 (55)	12 (60)
**Talking to the child, n (%)**								
	TS	36 (100)	37 (100)	36 (97)	31 (97)	30 (97)	23 (92)	19 (79)
	C	17 (47)	18 (49)	17 (47)	16 (52)	15 (50)	10 (44)	7 (37)
	I	19 (53)	19 (51)	19 (53)	15 (48)	15 (50)	13 (57)	12 (63)
**Eating/ having meals with the child, n (%)**								
	TS	34 (94)	34 (92)	32 (89)	29 (91)	30 (97)	22 (88)	20 (83)
	C	17 (50)	17 (50)	15 (47)	16 (55)	16 (53)	10 (46)	8 (40)
	I	17 (50)	17 (50)	17 (53)	13 (45)	14 (47)	12 (55)	12 (60)
**Physical contact with the child, n (%)**								
	TS	36 (100)	37 (100)	36 (100)	32 (100)	31 (100)	24 (96)	23 (96)
	C	17 (47)	18 (49)	17 (47)	17 (53)	16 (52)	11 (46)	9 (39)
	I	19 (53)	19 (51)	19 (53)	15 (47)	15 (48)	13 (54)	14 (61)

^a^Sample size varied for different categories and days due to nonresponses, ranging from 10 to 18 and from 13 to 21 in the control and intervention group, respectively.

^b^TS: total sample.

^c^C: control group.

^d^I: intervention group.

## Discussion

### Principal Findings

This is the first ECD randomized controlled trial testing an intervention using a smartphone-based ambulatory assessment to measure outcomes. The results showed that our nurse home visiting program had a positive effect on parental well-being and maternal parenting as measured by a smartphone eDiary. Previous studies have shown that maternal parenting behaviors in adolescent mothers [6] and low-income mothers [51,52] are less frequent. Consequently, it was surprising to notice a ceiling effect on some maternal parenting behaviors such as taking care of the child, talking to the child, and playing with the child, regardless of being part of the intervention or control group. Early childhood research specifically focused on maternal parenting is surprisingly lacking in Brazil, making it difficult to directly compare findings. Brazilian studies related to early life outcomes conducted in the past decade have mainly focused on maternal health [53] and older children [54]. However, we observed that the frequencies of telling stories or singing to the child and especially reading a book to the child were low overall (38%-62% and 11%-26%, respectively).

It is worth mentioning that Brazil is a country currently facing many educational challenges. The last report from the Programme for International Student Assessment (PISA) showed that reading performance among students has not improved in the last 18 years [55]. In the last PISA, Brazil and other Latin American countries ranked at the bottom of the international ranking on reading skills, below the top 50. Overall, 29% of the Brazilian population is functionally illiterate, presenting only basic reading and writing skills [56], and 44% of the Brazilian population have not fully or partially read a book in the last 3 months [57]. Telling stories and reading books are behaviors deeply connected to past behaviors and previous experiences. In a social context in which people have difficulties reading and understanding books, the ability and practice of telling stories may be impaired. Moreover, low-income families typically have less access to libraries [52] and usually cannot afford to buy books. Even though our intervention emphasized the importance of reading and telling stories, these barriers may have prevented participants from engaging in such activities.

We also found an intervention effect on daily reports of parental well-being. Parenting a child is known to have positive effects on adult parents such as higher levels of life satisfaction, happiness, general feeling of positive emotion, and more meaning in life [58]. However, adolescent mothers are at higher risk for maternal depression and impairment in parenting [6,59], and also experience more parental stress [60]. The effect of Primeiros Laços on parental well-being may be related to the parenting skills component of the intervention. After the child was born, the content of the program emphasized child development and early cognitive and socioemotional stimulation. During home visits, nurses showed the participants activities that were appropriate for the child’s age to enhance the mother-child bond and to provide adequate stimulation. We hypothesized that these specific activities would buffer the effects of parental stress and have a positive influence on parental well-being.

The compliance rate of the eDiary assessment was 84% (participants who responded at least one day of assessment), demonstrating that it was generally accepted by adolescent mothers living in an urban deprived area. This compliance rate was similar to that reported in other studies conducted with participants in the same age range. For example, a study conducted in Canada using a compliance criterion of participants responding to questions less than 7 times reported a rate of 90.4% [12]. A recent review on ecological momentary assessment studies with adolescents showed that among clinical studies with less than 3 assessments per day, the average compliance rate was 73.5%. This same review also showed that studies with more prompts per day (6 or more) had a higher compliance rate [61], suggesting that future studies should implement similar methods. Furthermore, a recent analysis of a pooled dataset from 10 ambulatory assessment studies comprising more than 1700 participants showed that compliance declined across days [62], which is similar to the pattern in the compliance rate observed in the present study. Even though a higher compliance rate across days would be ideal, we believe that our findings show that assessments conducted via smartphones can be adequately conducted in low-income areas. This method can be a low-cost alternative for clinical trials in LMICs, as well as a potential monitoring method linked to the public health system. In this way, health professionals could be notified when the frequency of parenting behaviors falls below the expectation to adequately stimulate child development, potentially leading to customized interventions.

### Limitations

Our findings should be viewed in light of some limitations. The sample size calculation of our clinical trial was based on different outcomes than those reported herein. The eDiary outcomes that were analyzed are secondary outcomes of the clinical trial, which were conceptualized and implemented after the clinical trial started. Therefore, our study may not have had sufficient power to find differences in these secondary outcomes, resulting in potential false negatives when compared to primary outcomes analyses. Additionally, our sample size limited the scope of our analyses. For instance, it would be interesting to use a structural equation modeling approach such as growth curves to validate the constructs assessed; however, the small sample sizes did not allow for convergence of these models. Moreover, the lack of variability and a ceiling effect in some variables may have also influenced these potential analyses. Therefore, even though we presented findings suggesting that we adequately measured the constructs of interest, we were not able to validate these measures quantitatively. However, we found that maternal parenting behaviors were negatively associated with maternal depression measured at the same time point [25], which is an expected finding since it is known that depressive symptoms can influence parenting. In addition, our assessment of maternal parenting was based exclusively on the frequency of behaviors, and we did not collect data on the quality of the interaction between the participants and their children. The quality of maternal parenting is known to be crucial for child development and the mother-child relationship. In addition, the use of eDiary self-report assessment may have influenced the findings due to effects of social desirability bias [63] or the participants’ awareness of being part of an ECD study [64]. However, this is unlikely since we detected low rates of some maternal parenting behaviors that would be considered socially approved. Moreover, we included a subgroup of participants who did not own a smartphone at the time of trial commencement. This was surprising since 86% of participants reported owning a smartphone at baseline. We found that it is a common practice among this population to frequently exchange, borrow, or buy smartphones. Furthermore, some participants owned old smartphones with limited hardware in terms of performance as well as with old operating systems, preventing them from properly using the smartphone app. We were able to include these participants by lending them a smartphone, which could have influenced our results. However, we did not find differences in outcomes between participants with their own smartphone compared with those who borrowed smartphones. Future implementations of smartphone-based assessments in low-income areas should consider that some people may not own a smartphone. This approach also may not be suited for rural areas, especially in LMICs, where poverty is more predominant.

### Conclusion

Our findings demonstrate the efficacy of Primeiros Laços, a nurse home visiting program for pregnant youth living in an urban deprived area, on improving maternal parenting and well-being assessed by an eDiary. Assessments with the eDiary were successfully conducted in this specific population. The implications are two-fold. First, Primeiros Laços is a promising intervention to promote maternal parenting and well-being among low-income adolescent mothers. As a structured and manualized intervention, this could be implemented in the primary care system, potentially benefiting millions of Brazilian mothers and children. Other LMICs similar to Brazil may also benefit from the program given appropriate adaptations for language, culture, and context. Second, our findings demonstrate the potential for future ECD intervention studies to implement ambulatory assessment in LMICs via smartphones for measuring mother and child behaviors. More frequent assessments of maternal parenting behaviors and well-being should be implemented to further enhance temporal and ecological validity, and to also expand the scope of measured behaviors.

## Appendix

Multimedia Appendix 1 CONSORT-eHEALTH checklist (V 1.6.1).

## Abbreviations

CONSORT: Consolidated Standards of Reporting Trials
ECD: early childhood development
eDiary: electronic daily diary
LMIC: low and middle-income country
PISA: Programme for International Student Assessment
