# Supplementary material for: Maternal Parenting Electronic Diary in the Context of a Home Visit Intervention for Adolescent Mothers in an Urban Deprived Area of São Paulo, Brazil: Randomized Controlled Trial
Source: JMIR Mhealth Uhealth. 2020 Jul 28;8(7):e13686. doi: 10.2196/13686 (PMC7420524; doi:10.2196/13686)
Supplement: Multimedia Appendix 1 [file mhealth_v8i7e13686_app1.pdf]

CONSORT-EHEALTH (V 1.6.1) - Submission/Publication Form

The CONSORT-EHEALTH checklist is intended for authors of randomized trials evaluating web-based and Internet-based applications/interventions, including mobile interventions, electronic games (incl multiplayer games), social media, certain telehealth applications, and other interactive and/or networked electronic applications. Some of the items (e.g. all subitems under item 5 - description of the intervention) may also be applicable for other study designs.

The goal of the CONSORT EHEALTH checklist and guideline is to be
a) a guide for reporting for authors of RCTs,
b) to form a basis for appraisal of an ehealth trial (in terms of validity)

CONSORT-EHEALTH items/subitems are MANDATORY reporting items for studies published in the Journal of Medical Internet Research and other journals / scientific societies endorsing the checklist.

Items numbered 1., 2., 3., 4a., 4b etc are original CONSORT or CONSORT-NPT (non-pharmacologic treatment) items.
Items with Roman numerals (i., ii, iii, iv etc.) are CONSORT-EHEALTH extensions/clarifications.

As the CONSORT-EHEALTH checklist is still considered in a formative stage, we would ask that you also RATE ON A SCALE OF 1-5 how important/useful you feel each item is FOR THE PURPOSE OF THE CHECKLIST and reporting guideline (optional).

Mandatory reporting items are marked with a red *.
In the textboxes, either copy & paste the relevant sections from your manuscript into this form - please include any quotes from your manuscript in QUOTATION MARKS,
or answer directly by providing additional information not in the manuscript, or elaborating on why the item was not relevant for this study.

YOUR ANSWERS WILL BE PUBLISHED AS A SUPPLEMENTARY FILE TO YOUR PUBLICATION IN JMIR AND ARE CONSIDERED PART OF YOUR PUBLICATION (IF ACCEPTED).
Please fill in these questions diligently. Information will not be copyedited, so please use proper spelling and grammar, use correct capitalization, and avoid abbreviations.

DO NOT FORGET TO SAVE AS PDF _AND_ CLICK THE SUBMIT BUTTON SO YOUR ANSWERS ARE IN OUR DATABASE !!!

Citation Suggestion (if you append the pdf as Appendix we suggest to cite this paper in the caption):
Eysenbach G, CONSORT-EHEALTH Group
CONSORT-EHEALTH: Improving and Standardizing Evaluation Reports of Web-based and

Mobile Health Interventions
J Med Internet Res 2011;13(4):e126
URL: http://www.jmir.org/2011/4/e126/
doi: 10.2196/jmir.1923
PMID: 22209829

* Required

Your name *

First Last

Daniel

Primary Affiliation (short), City, Country *

University of Toronto, Toronto, Canada

University of São Paulo

Your e-mail address *

abc@gmail.com

daniel.fatori@gmail.com

Title of your manuscript *

Provide the (draft) title of your manuscript.

Maternal Parenting Electronic Diary in the Context of a Home Visit Intervention for Adolescent Mothers in an Urban Deprived Area of São Paulo, Brazil: Randomized Controlled Trial

Name of your App/Software/Intervention *

If there is a short and a long/alternate name, write the short name first and add the long name in brackets.

Maternal Parenting Electronic Diary

Evaluated Version (if any)

e.g. "V1", "Release 2017-03-01", "Version 2.0.27913"

Your answer

Language(s) *

What language is the intervention/app in? If multiple languages are available, separate by comma (e.g. "English, French")

Brazilian Portuguese

URL of your Intervention Website or App

e.g. a direct link to the mobile app on app in appstore (itunes, Google Play), or URL of the website. If the intervention is a DVD or hardware, you can also link to an Amazon page.

Your answer

URL of an image/screenshot (optional)

Your answer

Accessibility *

Can an enduser access the intervention presently?

- ( ) access is free and open
- (x) access only for special usergroups, not open
- ( ) access is open to everyone, but requires payment/subscription/in-app purchases
- ( ) app/intervention no longer accessible
- ( ) Other:

Primary Medical Indication/Disease/Condition *

e.g. "Stress", "Diabetes", or define the target group in brackets after the condition, e.g. "Autism (Parents of children with)", "Alzheimers (Informal Caregivers of)"

Maternal Parenting, Well-being, Mother-child ir

Primary Outcomes measured in trial *

comma-separated list of primary outcomes reported in the trial

Maternal parenting, maternal well-being

Secondary/other outcomes

Are there any other outcomes the intervention is expected to affect?

Your answer

Recommended "Dose" *

What do the instructions for users say on how often the app should be used?

- [x] Approximately Daily
- [ ] Approximately Weekly
- [ ] Approximately Monthly
- [ ] Approximately Yearly
- [ ] "as needed"
- [ ] Other:

Approx. Percentage of Users (starters) still using the app as recommended after 3 months *

- [ ] unknown / not evaluated
- [ ] 0-10%
- [ ] 11-20%
- [ ] 21-30%
- [ ] 31-40%
- [ ] 41-50%
- [ ] 51-60%
- [ ] 61-70%
- [ ] 71%-80%
- [ ] 81-90%
- [ ] 91-100%
- [x] Other: Not applicable

Overall, was the app/intervention effective? *

- ( ) yes: all primary outcomes were significantly better in intervention group vs control
- (x) partly: SOME primary outcomes were significantly better in intervention group vs control
- ( ) no statistically significant difference between control and intervention
- ( ) potentially harmful: control was significantly better than intervention in one or more outcomes
- ( ) inconclusive: more research is needed
- ( ) Other:

Article Preparation Status/Stage *

At which stage in your article preparation are you currently (at the time you fill in this form)

- ( ) not submitted yet - in early draft status
- ( ) not submitted yet - in late draft status, just before submission
- ( ) submitted to a journal but not reviewed yet
- ( ) submitted to a journal and after receiving initial reviewer comments
- (x) submitted to a journal and accepted, but not published yet
- ( ) published
- ( ) Other:

Journal *

If you already know where you will submit this paper (or if it is already submitted), please provide the journal name (if it is not JMIR, provide the journal name under "other")

- ○ not submitted yet / unclear where I will submit this
- ○ Journal of Medical Internet Research (JMIR)
- ◉ JMIR mHealth and UHealth
- ○ JMIR Serious Games
- ○ JMIR Mental Health
- ○ JMIR Public Health
- ○ JMIR Formative Research
- ○ Other JMIR sister journal
- ○ Other:

Is this a full powered effectiveness trial or a pilot/feasibility trial? *

- ◉ Pilot/feasibility
- ○ Fully powered

Manuscript tracking number *

If this is a JMIR submission, please provide the manuscript tracking number under "other" (The ms tracking number can be found in the submission acknowledgement email, or when you login as author in JMIR. If the paper is already published in JMIR, then the ms tracking number is the four-digit number at the end of the DOI, to be found at the bottom of each published article in JMIR)

- ○ no ms number (yet) / not (yet) submitted to / published in JMIR
- ◉ Other: JMU ms#13686

TITLE AND ABSTRACT

1a) TITLE: Identification as a randomized trial in the title

1a) Does your paper address CONSORT item 1a? *

I.e does the title contain the phrase "Randomized Controlled Trial"? (if not, explain the reason under "other")

- (•) yes
- ( ) Other:

1a-i) Identify the mode of delivery in the title

Identify the mode of delivery. Preferably use “web-based” and/or “mobile” and/or “electronic game” in the title. Avoid ambiguous terms like “online”, “virtual”, “interactive”. Use “Internet-based” only if Intervention includes non-web-based Internet components (e.g. email), use “computer-based” or “electronic” only if offline products are used. Use “virtual” only in the context of “virtual reality” (3-D worlds). Use “online” only in the context of “online support groups”. Complement or substitute product names with broader terms for the class of products (such as “mobile” or “smart phone” instead of “iphone”), especially if the application runs on different platforms.

	1	2	3	4	5	
subitem not at all important	( )	( )	(•)	( )	( )	essential

Does your paper address subitem 1a-i? *

Copy and paste relevant sections from manuscript title (include quotes in quotation marks "like this" to indicate direct quotes from your manuscript), or elaborate on this item by providing additional information not in the ms, or briefly explain why the item is not applicable/relevant for your study

"Parenting Electronic Diary Home Visit Intervention for Adolescent Mothers".

1a-ii) Non-web-based components or important co-interventions in title

Mention non-web-based components or important co-interventions in title, if any (e.g., “with telephone support”).

	1	2	3	4	5	
subitem not at all important	○	○	○	○	◉	essential

Does your paper address subitem 1a-ii?

Copy and paste relevant sections from manuscript title (include quotes in quotation marks "like this" to indicate direct quotes from your manuscript), or elaborate on this item by providing additional information not in the ms, or briefly explain why the item is not applicable/relevant for your study

"The present study is part of a randomized controlled trial that was originally designed to test the efficacy of Primeiros Laços, a nurse home-visiting program for adolescent mothers living in an urban deprived area of São Paulo, Brazil".

1a-iii) Primary condition or target group in the title

Mention primary condition or target group in the title, if any (e.g., “for children with Type I Diabetes”) Example: A Web-based and Mobile Intervention with Telephone Support for Children with Type I Diabetes: Randomized Controlled Trial

	1	2	3	4	5	
subitem not at all important	○	○	○	◉	○	essential

Does your paper address subitem 1a-iii? *

Copy and paste relevant sections from manuscript title (include quotes in quotation marks "like this" to indicate direct quotes from your manuscript), or elaborate on this item by providing additional information not in the ms, or briefly explain why the item is not applicable/relevant for your study

"A total of 169 pregnant youth were assessed for eligibility criteria, 80 of whom were included and randomized to the intervention (n=40) and control (care as usual) group (n=40). Inclusion criteria were: (a) aged 14 to 19 years old, (b) first pregnancy, (c) between 8-16 weeks of gestation, (d) low socioeconomic status (classes C, D, E according to the widely used Brazilian ABEP scale)".

1b) ABSTRACT: Structured summary of trial design, methods, results, and conclusions

NPT extension: Description of experimental treatment, comparator, care providers, centers, and blinding status.

1b-i) Key features/functionalities/components of the intervention and comparator in the METHODS section of the ABSTRACT

Mention key features/functionalities/components of the intervention and comparator in the abstract. If possible, also mention theories and principles used for designing the site. Keep in mind the needs of systematic reviewers and indexers by including important synonyms. (Note: Only report in the abstract what the main paper is reporting. If this information is missing from the main body of text, consider adding it)

	1	2	3	4	5	
subitem not at all important	○	○	○	○	○	essential

Does your paper address subitem 1b-i? *

Copy and paste relevant sections from the manuscript abstract (include quotes in quotation marks "like this" to indicate direct quotes from your manuscript), or elaborate on this item by providing additional information not in the ms, or briefly explain why the item is not applicable/relevant for your study

"Primeiros Laços is a home visiting intervention delivered by trained nurses tailored to first-time pregnant adolescents and their children, starting during the first 16 weeks of pregnancy until the child reaches 24 months of age. (...) At 18 months, participants were assessed regarding maternal parenting and parental well-being using a 7-consecutive-day eDiary. The smartphone app was programmed to notify participants every day at 9:00 PM over a period of seven days".

1b-ii) Level of human involvement in the METHODS section of the ABSTRACT

Clarify the level of human involvement in the abstract, e.g., use phrases like “fully automated” vs. “therapist/nurse/care provider/physician-assisted” (mention number and expertise of providers involved, if any). (Note: Only report in the abstract what the main paper is reporting. If this information is missing from the main body of text, consider adding it)

	1	2	3	4	5	
subitem not at all important	○	○	○	○	◉	essential

Does your paper address subitem 1b-ii?

Copy and paste relevant sections from the manuscript abstract (include quotes in quotation marks "like this" to indicate direct quotes from your manuscript), or elaborate on this item by providing additional information not in the ms, or briefly explain why the item is not applicable/relevant for your study

"Primeiros Laços is a home visiting intervention delivered by trained nurses tailored to first-time pregnant adolescents and their children. (...) Participants were assessed by blind interviewers at 8-16 weeks of pregnancy (baseline), 30 weeks of pregnancy, and when the child was 3, 6, and 12 months of age. At 18 months, participants were assessed regarding maternal parenting and parental well-being using a 7-consecutive-day eDiary. The smartphone app was programmed to notify participants every day at 9:00 PM over a period of seven days".

1b-iii) Open vs. closed, web-based (self-assessment) vs. face-to-face assessments in the METHODS section of the ABSTRACT

Mention how participants were recruited (online vs. offline), e.g., from an open access website or from a clinic or a closed online user group (closed usergroup trial), and clarify if this was a purely web-based trial, or there were face-to-face components (as part of the intervention or for assessment). Clearly say if outcomes were self-assessed through questionnaires (as common in web-based trials). Note: In traditional offline trials, an open trial (open-label trial) is a type of clinical trial in which both the researchers and participants know which treatment is being administered. To avoid confusion, use “blinded” or “unblinded” to indicated the level of blinding instead of “open”, as “open” in web-based trials usually refers to “open access” (i.e. participants can self-enrol). (Note: Only report in the abstract what the main paper is reporting. If this information is missing from the main body of text, consider adding it)

	1	2	3	4	5	
subitem not at all important	○	○	◉	○	○	essential

Does your paper address subitem 1b-iii?

Copy and paste relevant sections from the manuscript abstract (include quotes in quotation marks "like this" to indicate direct quotes from your manuscript), or elaborate on this item by providing additional information not in the ms, or briefly explain why the item is not applicable/relevant for your study

"A total of 169 pregnant adolescents were assessed for eligibility criteria, 80 of whom were included and randomized to the intervention (n=40) and control group (care as usual, n=40). Primeiros Laços is a home visiting intervention delivered by trained nurses tailored to first-time pregnant adolescents and their children, starting during the first 16 weeks of pregnancy until the child reaches 24 months of age. Participants were assessed by blind interviewers at 8-16 weeks of pregnancy (baseline), 30 weeks of pregnancy, and when the child was 3, 6, and 12 months of age. At 18 months, participants were assessed regarding maternal parenting and parental well-being using a 7-consecutive-day eDiary. The smartphone app was programmed to notify participants every day at 9:00 PM over a period of seven days".

1b-iv) RESULTS section in abstract must contain use data

Report number of participants enrolled/assessed in each group, the use/uptake of the intervention (e.g., attrition/adherence metrics, use over time, number of logins etc.), in addition to primary/secondary outcomes. (Note: Only report in the abstract what the main paper is reporting. If this information is missing from the main body of text, consider adding it)

	1	2	3	4	5	
subitem not at all important	○	○	◉	○	○	essential

Does your paper address subitem 1b-iv?

Copy and paste relevant sections from the manuscript abstract (include quotes in quotation marks "like this" to indicate direct quotes from your manuscript), or elaborate on this item by providing additional information not in the ms, or briefly explain why the item is not applicable/relevant for your study

"We were able to contact 57/80 (71%) participants (29 from the intervention group and 28 from the control group) when the child was 18 months of age. Forty-eight of the 57 participants (84%) completed at least one day of the eDiary protocol. The daily compliance rate ranged from 49% to 70%. Our analyses showed a significant effect of the intervention on parental well-being (B=0.32, P=.02) and the maternal parenting behavior of the mother telling a story or singing to the child (odds ratio=2.33, P=.01)".

1b-v) CONCLUSIONS/DISCUSSION in abstract for negative trials

Conclusions/Discussions in abstract for negative trials: Discuss the primary outcome - if the trial is negative (primary outcome not changed), and the intervention was not used, discuss whether negative results are attributable to lack of uptake and discuss reasons. (Note: Only report in the abstract what the main paper is reporting. If this information is missing from the main body of text, consider adding it)

	1	2	3	4	5	
subitem not at all important	(●)	( )	( )	( )	( )	essential

Does your paper address subitem 1b-v?

Copy and paste relevant sections from the manuscript abstract (include quotes in quotation marks "like this" to indicate direct quotes from your manuscript), or elaborate on this item by providing additional information not in the ms, or briefly explain why the item is not applicable/relevant for your study

Not applicable.

INTRODUCTION

2a) In INTRODUCTION: Scientific background and explanation of rationale

2a-i) Problem and the type of system/solution

Describe the problem and the type of system/solution that is object of the study: intended as stand-alone intervention vs. incorporated in broader health care program? Intended for a particular patient population? Goals of the intervention, e.g., being more cost-effective to other interventions, replace or complement other solutions? (Note: Details about the intervention are provided in “Methods” under 5)

	1	2	3	4	5	
subitem not at all important	( )	( )	( )	(●)	( )	essential

Does your paper address subitem 2a-i? *

Copy and paste relevant sections from the manuscript (include quotes in quotation marks "like this" to indicate direct quotes from your manuscript), or elaborate on this item by providing additional information not in the ms, or briefly explain why the item is not applicable/relevant for your study

"Studies focusing on maternal parenting usually rely on direct or recorded observation of dyads by trained and certified experts [8] or self-reported questionnaires based on retrospective behavior. However, these methods have important limitations. Observation of dyads is a lengthy and costly assessment, which is difficult to implement in contexts where resources (eg, trained experts, available space) are scarce and accessibility is limited, such as in LMICs. Retrospective self-reported questionnaires are subject to memory bias and lack ecological validity, resulting in potentially inaccurate data (information bias). The use of ambulatory assessment delivered via smartphones can be an alternative data collection method to circumvent these problems".

"Ambulatory assessment encompasses methods designed to study people in their natural environment. Owing to the ubiquitous presence of smartphones, ambulatory assessment methods are now most commonly delivered via mobile technology. Subjects can be notified to answer questions during specific times of the day throughout days, weeks, or months. Given the numerous advantages of ambulatory assessment, its use has recently grown in many fields [9-11]. Nevertheless, the field of ECD has been slow in adopting ambulatory assessment, with few initiatives conducted in developed countries"

2a-ii) Scientific background, rationale: What is known about the (type of) system

Scientific background, rationale: What is known about the (type of) system that is the object of the study (be sure to discuss the use of similar systems for other conditions/diagnoses, if appropiate), motivation for the study, i.e. what are the reasons for and what is the context for this specific study, from which stakeholder viewpoint is the study performed, potential impact of findings [2]. Briefly justify the choice of the comparator.

	1	2	3	4	5	
subitem not at all important	○	○	◉	○	○	essential

Does your paper address subitem 2a-ii? *

Copy and paste relevant sections from the manuscript (include quotes in quotation marks "like this" to indicate direct quotes from your manuscript), or elaborate on this item by providing additional information not in the ms, or briefly explain why the item is not applicable/relevant for your study

"Ambulatory assessment encompasses methods designed to study people in their natural environment. Owing to the ubiquitous presence of smartphones, ambulatory assessment methods are now most commonly delivered via mobile technology. Subjects can be notified to answer questions during specific times of the day throughout days, weeks, or months. Given the numerous advantages of ambulatory assessment, its use has recently grown in many fields [9-11]. Nevertheless, the field of ECD has been slow in adopting ambulatory assessment, with few initiatives conducted in developed countries. One study used the ambulatory assessment method to assess the relationship between crying and fussing at 12 months of age along with the physical health of the child and emotional security of the mother [12]. Another study validated an ambulatory assessment protocol to evaluate parental discipline in children aged 18-36 months [13]. However, to our knowledge, no study conducted in an LMIC has tested the potential of ambulatory assessment in the context of ECD programs. Moreover, ambulatory assessment methods have only been adopted in observational studies to date, and there has been no randomized trial in the ECD field that used ambulatory assessment via mobile technology as an outcome. Most studies testing mobile technology in the field of ECD are interventions related to various outcomes such as maternal depression [14], infant feeding [15-17], physical activity [18], and maternal health [19-22]"

2b) In INTRODUCTION: Specific objectives or hypotheses

Does your paper address CONSORT subitem 2b? *

Copy and paste relevant sections from the manuscript (include quotes in quotation marks "like this" to indicate direct quotes from your manuscript), or elaborate on this item by providing additional information not in the ms, or briefly explain why the item is not applicable/relevant for your study

"Therefore, we implemented a randomized controlled study to test the effects of an electronic daily diary (eDiary) as a specific type of ambulatory assessment through which participants report a set of behaviors that occurred during the day to measure maternal parenting and parental well-being. In particular, we assessed the efficacy of Primeiros Laços, a nurse home-visiting program for adolescent mothers living in an urban deprived area of São Paulo, Brazil. The intervention aimed to foster the mother-child relationship and improve child development. The present study had two objectives: (1) to test the efficacy of a nurse home visiting intervention on child maternal parenting and parental well-being measured by an eDiary, and (2) to investigate the compliance rate of the eDiary measurement method. The first hypothesis was that mothers who receive the home visiting intervention will present a higher frequency of maternal parenting behaviors and higher scores of well-being. The second hypothesis was that the eDiary method will present a compliance rate similar to that reported in previous studies, despite the fact that our sample is composed of low-income adolescent mothers living in adverse

METHODS

3a) Description of trial design (such as parallel, factorial) including allocation ratio

Does your paper address CONSORT subitem 3a? *

Copy and paste relevant sections from the manuscript (include quotes in quotation marks "like this" to indicate direct quotes from your manuscript), or elaborate on this item by providing additional information not in the ms, or briefly explain why the item is not applicable/relevant for your study

"The present study is part of a parallel group randomized controlled trial that was originally designed to test the efficacy of Primeiros Laços".

"The allocation ratio was 1:1."

3b) Important changes to methods after trial commencement (such as eligibility criteria), with reasons

Does your paper address CONSORT subitem 3b? *

Copy and paste relevant sections from the manuscript (include quotes in quotation marks "like this" to indicate direct quotes from your manuscript), or elaborate on this item by providing additional information not in the ms, or briefly explain why the item is not applicable/relevant for your study

"The eDiary outcomes that were analyzed are secondary outcomes of the clinical trial, which were conceptualized and implemented after the clinical trial started. Therefore, our study may not have had sufficient power to find differences in these secondary outcomes, resulting in potential false negatives when compared to primary outcomes

3b-i) Bug fixes, Downtimes, Content Changes

Bug fixes, Downtimes, Content Changes: ehealth systems are often dynamic systems. A description of changes to methods therefore also includes important changes made on the intervention or comparator during the trial (e.g., major bug fixes or changes in the functionality or content) (5-iii) and other “unexpected events” that may have influenced study design such as staff changes, system failures/downtimes, etc. [2].

	1	2	3	4	5	
subitem not at all important	◉	○	○	○	○	essential

Does your paper address subitem 3b-i?

Copy and paste relevant sections from the manuscript (include quotes in quotation marks "like this" to indicate direct quotes from your manuscript), or elaborate on this item by providing additional information not in the ms, or briefly explain why the item is not applicable/relevant for your study

No changes were made to the app during or after the trial.

4a) Eligibility criteria for participants

Does your paper address CONSORT subitem 4a? *

Copy and paste relevant sections from the manuscript (include quotes in quotation marks "like this" to indicate direct quotes from your manuscript), or elaborate on this item by providing additional information not in the ms, or briefly explain why the item is not applicable/relevant for your study

"Inclusion criteria were: (a) aged 14 to 19 years old, (b) first pregnancy, (c) between 8-16 weeks of gestation, (d) low socioeconomic status (classes C, D, E according to the widely used Brazilian ABEP scale) [27], and (e) living in the western region of the city of Sao Paulo."

4a-i) Computer / Internet literacy

Computer / Internet literacy is often an implicit "de facto" eligibility criterion - this should be explicitly clarified.

	1	2	3	4	5	
subitem not at all important	○	○	○	●	○	essential

Does your paper address subitem 4a-i?

Copy and paste relevant sections from the manuscript (include quotes in quotation marks "like this" to indicate direct quotes from your manuscript), or elaborate on this item by providing additional information not in the ms, or briefly explain why the item is not applicable/relevant for your study

"All participants were familiar with smartphones and internet use."

4a-ii) Open vs. closed, web-based vs. face-to-face assessments:

Open vs. closed, web-based vs. face-to-face assessments: Mention how participants were recruited (online vs. offline), e.g., from an open access website or from a clinic, and clarify if this was a purely web-based trial, or there were face-to-face components (as part of the intervention or for assessment), i.e., to what degree got the study team to know the participant. In online-only trials, clarify if participants were quasi-anonymous and whether having multiple identities was possible or whether technical or logistical measures (e.g., cookies, email confirmation, phone calls) were used to detect/prevent these.

	1	2	3	4	5	
subitem not at all important	○	○	○	○	●	essential

Does your paper address subitem 4a-ii? *

Copy and paste relevant sections from the manuscript (include quotes in quotation marks "like this" to indicate direct quotes from your manuscript), or elaborate on this item by providing additional information not in the ms, or briefly explain why the item is not applicable/relevant for your study

"Participants were recruited in the primary health care system." (offline recruitment).

Intervention was face-to-face: "Primeiros Laços is a home visiting intervention delivered by trained nurses that is tailored to first-time adolescent pregnant women and their children, which starts during the first 16 weeks of pregnancy until the child reaches 24 months of age".

Assessments were conducted partially face-to-face: "Participants were assessed by interviewers blinded to group allocation at 8-16 weeks of pregnancy (baseline), 30 weeks of pregnancy, and when the child was 3, 6, 12, 18, and 24 months of age. Interviews were conducted by trained psychologists who underwent a 1-month training program provided by senior psychiatrists, psychologists, and pediatricians. Before study interviews commenced, interviewers trained by assessing volunteers living in the western region of Sao Paulo. Ambulatory assessment was administered when the child was 18 months old. At baseline (8-16 weeks of pregnancy), we measured depression symptoms using the Beck Depression Inventory [41,42] and anxiety symptoms were assessed using the Beck Anxiety Inventory [43,44], which have both been validated in Brazil. Family food insecurity was measured using the abridged version of the Brazilian Food Insecurity Scale (Escala de Insegurança Alimentar) [45], a widely used scale adopted in national epidemiological studies [46]".

eDiary outcomes happened via smartphone app: "We used the LifeData system [47], a mobile technology suite for smartphone secure data collection available for Android and iOS. Our eDiary protocol comprised a set of questions designed via a web-based dashboard. The smartphone app was programmed to notify participants every day at 9:00 PM over a period of 7 days. Participants had up to 120 minutes to complete the eDiary protocol and received up to three reminders. Trained psychologists visited participants to provide support on downloading, installing, and using the app. The eDiary protocol was shown and explained to all participants using a demonstration version installed on the psychologist's smartphone. Participants who did not own a smartphone were provided one for the duration of the eDiary protocol (n=18). All participants were familiar with smartphones and internet use."

4a-iii) Information giving during recruitment

Information given during recruitment. Specify how participants were briefed for recruitment and in the informed consent procedures (e.g., publish the informed consent documentation as appendix, see also item X26), as this information may have an effect on user self-selection, user expectation and may also bias results.

	1	2	3	4	5	
subitem not at all important	○	○	○	◉	○	essential

Does your paper address subitem 4a-iii?

Copy and paste relevant sections from the manuscript (include quotes in quotation marks "like this" to indicate direct quotes from your manuscript), or elaborate on this item by providing additional information not in the ms, or briefly explain why the item is not applicable/relevant for your study

"Trained psychologists visited participants to provide support on downloading, installing, and using the app. The eDiary protocol was shown and explained to all participants using a demonstration version installed on the psychologist's smartphone. Participants who did not own a smartphone were provided one for the duration of the eDiary protocol (n=18). All participants were familiar with smartphones and internet use."

4b) Settings and locations where the data were collected

Does your paper address CONSORT subitem 4b? *

Copy and paste relevant sections from the manuscript (include quotes in quotation marks "like this" to indicate direct quotes from your manuscript), or elaborate on this item by providing additional information not in the ms, or briefly explain why the item is not applicable/relevant for your study

"The present study is part of a parallel group randomized controlled trial that was originally designed to test the efficacy of Primeiros Laços, a nurse home-visiting program for adolescent mothers living in an urban deprived area of São Paulo, Brazil. Previous findings from this trial can be found elsewhere [23-26]. A total of 169 pregnant youth were assessed for eligibility criteria, 80 of whom were included and randomized to the intervention (n=40) and control (care as usual) group (n=40). Inclusion criteria were: (a) aged 14 to 19 years old, (b) first pregnancy, (c) between 8-16 weeks of gestation, (d) low socioeconomic status (classes C, D, E according to the widely used Brazilian ABEP scale) [27], and (e) living in the western region of the city of Sao Paulo."

4b-i) Report if outcomes were (self-)assessed through online questionnaires

Clearly report if outcomes were (self-)assessed through online questionnaires (as common in web-based trials) or otherwise.

	1	2	3	4	5	
subitem not at all important	○	○	○	○	○	essential

Does your paper address subitem 4b-i? *

Copy and paste relevant sections from the manuscript (include quotes in quotation marks "like this" to indicate direct quotes from your manuscript), or elaborate on this item by providing additional information not in the ms, or briefly explain why the item is not applicable/relevant for your study

Outcomes were reported via a smartphone app (self-assessment): "Our eDiary protocol comprised a set of questions designed via a web-based dashboard. The smartphone app was programmed to notify participants every day at 9:00 PM over a period of 7 days. Participants had up to 120 minutes to complete the eDiary protocol and received up to three reminders."

4b-ii) Report how institutional affiliations are displayed

Report how institutional affiliations are displayed to potential participants [on ehealth media], as affiliations with prestigious hospitals or universities may affect volunteer rates, use, and reactions with regards to an intervention.(Not a required item – describe only if this may bias results)

	1	2	3	4	5	
subitem not at all important	○	○	○	○	○	essential

Does your paper address subitem 4b-ii?

Copy and paste relevant sections from the manuscript (include quotes in quotation marks "like this" to indicate direct quotes from your manuscript), or elaborate on this item by providing additional information not in the ms, or briefly explain why the item is not applicable/relevant for your study

Institutional affiliations were not displayed in the smartphone app. But trained psychologists explained about the app and participants were enrolled in the trial for approximately 2 years at the time of this study, meaning that participants were well aware of institutional affiliations.

"Trained psychologists visited participants to provide support on downloading, installing, and using the app. The eDiary protocol was shown and explained to all participants using a demonstration version installed on the psychologist's smartphone."

5) The interventions for each group with sufficient details to allow replication, including how and when they were actually administered

5-i) Mention names, credential, affiliations of the developers, sponsors, and owners

Mention names, credential, affiliations of the developers, sponsors, and owners [6] (if authors/evaluators are owners or developer of the software, this needs to be declared in a "Conflict of interest" section or mentioned elsewhere in the manuscript).

	1	2	3	4	5	
subitem not at all important	○	○	●	○	○	essential

Does your paper address subitem 5-i?

Copy and paste relevant sections from the manuscript (include quotes in quotation marks "like this" to indicate direct quotes from your manuscript), or elaborate on this item by providing additional information not in the ms, or briefly explain why the item is not applicable/relevant for your study

"We used the LifeData system [47], a mobile technology suite for smartphone secure data collection available for Android and iOS."

5-ii) Describe the history/development process

Describe the history/development process of the application and previous formative evaluations (e.g., focus groups, usability testing), as these will have an impact on adoption/use rates and help with interpreting results.

	1	2	3	4	5	
subitem not at all important	(•)	( )	( )	( )	( )	essential

Does your paper address subitem 5-ii?

Copy and paste relevant sections from the manuscript (include quotes in quotation marks "like this" to indicate direct quotes from your manuscript), or elaborate on this item by providing additional information not in the ms, or briefly explain why the item is not applicable/relevant for your study

We did not develop the smartphone app used in the study.

5-iii) Revisions and updating

Revisions and updating. Clearly mention the date and/or version number of the application/intervention (and comparator, if applicable) evaluated, or describe whether the intervention underwent major changes during the evaluation process, or whether the development and/or content was “frozen” during the trial. Describe dynamic components such as news feeds or changing content which may have an impact on the replicability of the intervention (for unexpected events see item 3b).

	1	2	3	4	5	
subitem not at all important	(•)	( )	( )	( )	( )	essential

Does your paper address subitem 5-iii?

Copy and paste relevant sections from the manuscript (include quotes in quotation marks "like this" to indicate direct quotes from your manuscript), or elaborate on this item by providing additional information not in the ms, or briefly explain why the item is not applicable/relevant for your study

No updates or revisions were made during or after the trial.

5-iv) Quality assurance methods

Provide information on quality assurance methods to ensure accuracy and quality of information provided [1], if applicable.

	1	2	3	4	5	
subitem not at all important	(•)	( )	( )	( )	( )	essential

Does your paper address subitem 5-iv?

Copy and paste relevant sections from the manuscript (include quotes in quotation marks "like this" to indicate direct quotes from your manuscript), or elaborate on this item by providing additional information not in the ms, or briefly explain why the item is not applicable/relevant for your study

Not applicable. No information were provided by the smartphone app. The app only function was to provide self-assessment to participants.

5-v) Ensure replicability by publishing the source code, and/or providing screenshots/screen-capture video, and/or providing flowcharts of the algorithms used

Ensure replicability by publishing the source code, and/or providing screenshots/screen-capture video, and/or providing flowcharts of the algorithms used. Replicability (i.e., other researchers should in principle be able to replicate the study) is a hallmark of scientific reporting.

	1	2	3	4	5	
subitem not at all important	( )	( )	( )	( )	( )	essential

Does your paper address subitem 5-v?

Copy and paste relevant sections from the manuscript (include quotes in quotation marks "like this" to indicate direct quotes from your manuscript), or elaborate on this item by providing additional information not in the ms, or briefly explain why the item is not applicable/relevant for your study

We did not develop the smartphone app used in the study, therefore we cannot provide the source code. More information can be found in www.lifedatacorp.com.

All questions of the self-assessment relevant to the study are mentioned in the manuscript (see below).

"The present study focused on the following questions of the eDiary protocol: (1) How do you feel about the day today? (score 1-100, general maternal well-being) (2) Did you take care of your child or spend some time with him/her today? (3) What was it like taking care of your child today? (score 1-100, parental well-being) (4) Did you read or show a book to your child today?; (5) Did you tell stories or sing to your child today? (6) Did you go out or go for a stroll with your child today? (7) Did you play with your child today? (8) Did you talk to your child today? (9) Did you eat or have a meal with your child today? (10) Did you kiss, hug, tickle (or have any other physical contact with) your child today? (11) How many hours did you spend with your child today? Maternal parenting behavior was operationalized as parenting practices related to mother-child interaction and positive activities known to stimulate child development. Questions 2, and 4 to 11 were related to maternal parenting."

5-vi) Digital preservation

Digital preservation: Provide the URL of the application, but as the intervention is likely to change or disappear over the course of the years; also make sure the intervention is archived (Internet Archive, webcitation.org, and/or publishing the source code or screenshots/videos alongside the article). As pages behind login screens cannot be archived, consider creating demo pages which are accessible without login.

	1	2	3	4	5	
subitem not at all important	◉	○	○	○	○	essential

Does your paper address subitem 5-vi?

Copy and paste relevant sections from the manuscript (include quotes in quotation marks "like this" to indicate direct quotes from your manuscript), or elaborate on this item by providing additional information not in the ms, or briefly explain why the item is not applicable/relevant for your study

Screenshots of the smartphone assessment can be found in imgur.com/a/eTMAp84

"Screenshots of the eDiary protocol can be found elsewhere (imgur.com/a/eTMAp84)."

5-vii) Access

Access: Describe how participants accessed the application, in what setting/context, if they had to pay (or were paid) or not, whether they had to be a member of specific group. If known, describe how participants obtained "access to the platform and Internet" [1]. To ensure access for editors/reviewers/readers, consider to provide a "backdoor" login account or demo mode for reviewers/readers to explore the application (also important for archiving purposes, see vi).

	1	2	3	4	5	
subitem not at all important	○	○	○	◉	○	essential

Does your paper address subitem 5-vii? *

Copy and paste relevant sections from the manuscript (include quotes in quotation marks "like this" to indicate direct quotes from your manuscript), or elaborate on this item by providing additional information not in the ms, or briefly explain why the item is not applicable/relevant for your study

"Trained psychologists visited participants to provide support on downloading, installing, and using the app. The eDiary protocol was shown and explained to all participants using a demonstration version installed on the psychologist's smartphone. Participants who did not own a smartphone were provided one for the duration of the eDiary protocol (n=18)."

5-viii) Mode of delivery, features/functionalities/components of the intervention and comparator, and the theoretical framework

Describe mode of delivery, features/functionalities/components of the intervention and comparator, and the theoretical framework [6] used to design them (instructional strategy [1], behaviour change techniques, persuasive features, etc., see e.g., [7, 8] for terminology). This includes an in-depth description of the content (including where it is coming from and who developed it) [1]," whether [and how] it is tailored to individual circumstances and allows users to track their progress and receive feedback" [6]. This also includes a description of communication delivery channels and – if computer-mediated communication is a component – whether communication was synchronous or asynchronous [6]. It also includes information on presentation strategies [1], including page design principles, average amount of text on pages, presence of hyperlinks to other resources, etc. [1].

	1	2	3	4	5	
subitem not at all important	(•)	( )	( )	( )	( )	essential

Does your paper address subitem 5-viii? *

Copy and paste relevant sections from the manuscript (include quotes in quotation marks "like this" to indicate direct quotes from your manuscript), or elaborate on this item by providing additional information not in the ms, or briefly explain why the item is not applicable/relevant for your study

Not applicable. The smartphone app described here was used only to assess outcomes of a randomized controlled trial. The intervention was a nurse home visiting program.

5-ix) Describe use parameters

Describe use parameters (e.g., intended "doses" and optimal timing for use). Clarify what instructions or recommendations were given to the user, e.g., regarding timing, frequency, heaviness of use, if any, or was the intervention used ad libitum.

	1	2	3	4	5	
subitem not at all important	( )	( )	( )	(•)	( )	essential

Does your paper address subitem 5-ix?

Copy and paste relevant sections from the manuscript (include quotes in quotation marks "like this" to indicate direct quotes from your manuscript), or elaborate on this item by providing additional information not in the ms, or briefly explain why the item is not applicable/relevant for your study

"The smartphone app was programmed to notify participants every day at 9:00 PM over a period of 7 days. Participants had up to 120 minutes to complete the eDiary protocol and received up to three reminders."

5-x) Clarify the level of human involvement

Clarify the level of human involvement (care providers or health professionals, also technical assistance) in the e-intervention or as co-intervention (detail number and expertise of professionals involved, if any, as well as “type of assistance offered, the timing and frequency of the support, how it is initiated, and the medium by which the assistance is delivered”. It may be necessary to distinguish between the level of human involvement required for the trial, and the level of human involvement required for a routine application outside of a RCT setting (discuss under item 21 – generalizability).

	1	2	3	4	5	
subitem not at all important	○	○	○	◉	○	essential

Does your paper address subitem 5-x?

Copy and paste relevant sections from the manuscript (include quotes in quotation marks "like this" to indicate direct quotes from your manuscript), or elaborate on this item by providing additional information not in the ms, or briefly explain why the item is not applicable/relevant for your study

"The present study is part of a parallel group randomized controlled trial that was originally designed to test the efficacy of Primeiros Laços, a nurse home-visiting program for adolescent mothers living in an urban deprived area of São Paulo, Brazil."

"Primeiros Laços is a home visiting intervention delivered by trained nurses that is tailored to first-time adolescent pregnant women and their children, which starts during the first 16 weeks of pregnancy until the child reaches 24 months of age. The frequency of visits was (a) biweekly during gestation and from 2 to 20 months of the child's age; (b) weekly during the first and last month of pregnancy, and puerperium; and (c) monthly from 21 to 24 months of the child's age."

"Participants were assessed by interviewers blinded to group allocation at 8-16 weeks of pregnancy (baseline), 30 weeks of pregnancy, and when the child was 3, 6, 12, 18, and 24 months of age."

"Trained psychologists visited participants to provide support on downloading, installing, and using the app. The eDiary protocol was shown and explained to all participants using a demonstration version installed on the psychologist's smartphone"

5-xi) Report any prompts/reminders used

Report any prompts/reminders used: Clarify if there were prompts (letters, emails, phone calls, SMS) to use the application, what triggered them, frequency etc. It may be necessary to distinguish between the level of prompts/reminders required for the trial, and the level of prompts/reminders for a routine application outside of a RCT setting (discuss under item 21 – generalizability).

	1	2	3	4	5	
subitem not at all important	○	○	◉ (selected)	○	○	essential

Does your paper address subitem 5-xi? *

Copy and paste relevant sections from the manuscript (include quotes in quotation marks "like this" to indicate direct quotes from your manuscript), or elaborate on this item by providing additional information not in the ms, or briefly explain why the item is not applicable/relevant for your study

"The smartphone app was programmed to notify participants every day at 9:00 PM over a period of 7 days. Participants had up to 120 minutes to complete the eDiary protocol and received up to three reminders."

5-xii) Describe any co-interventions (incl. training/support)

Describe any co-interventions (incl. training/support): Clearly state any interventions that are provided in addition to the targeted eHealth intervention, as ehealth intervention may not be designed as stand-alone intervention. This includes training sessions and support [1]. It may be necessary to distinguish between the level of training required for the trial, and the level of training for a routine application outside of a RCT setting (discuss under item 21 – generalizability.

	1	2	3	4	5	
subitem not at all important	○	○	○	○	○	essential

Does your paper address subitem 5-xii? *

Copy and paste relevant sections from the manuscript (include quotes in quotation marks "like this" to indicate direct quotes from your manuscript), or elaborate on this item by providing additional information not in the ms, or briefly explain why the item is not applicable/relevant for your study

"The present study is part of a parallel group randomized controlled trial that was originally designed to test the efficacy of Primeiros Laços, a nurse home-visiting program for adolescent mothers living in an urban deprived area of São Paulo, Brazil."

"Primeiros Laços is a home visiting intervention delivered by trained nurses that is tailored to first-time adolescent pregnant women and their children, which starts during the first 16 weeks of pregnancy until the child reaches 24 months of age. The frequency of visits was (a) biweekly during gestation and from 2 to 20 months of the child's age; (b) weekly during the first and last month of pregnancy, and puerperium; and (c) monthly from 21 to 24 months of the child's age."

6a) Completely defined pre-specified primary and secondary outcome measures, including how and when they were assessed

Does your paper address CONSORT subitem 6a? *

Copy and paste relevant sections from the manuscript (include quotes in quotation marks "like this" to indicate direct quotes from your manuscript), or elaborate on this item by providing additional information not in the ms, or briefly explain why the item is not applicable/relevant for your study

The present study analysed secondary outcomes of an ongoing trial designed to test the efficacy of a home visiting program on electroencephalogram alpha wave frequency and child development measured by the Bayley Scales of Infant Development.

We address this issue in the Limitations paragraph:

"The eDiary outcomes that were analyzed are secondary outcomes of the clinical trial, which were conceptualized and implemented after the clinical trial started. Therefore, our study may not have had sufficient power to find differences in these secondary outcomes, resulting in potential false negatives when compared to primary outcomes

6a-i) Online questionnaires: describe if they were validated for online use and apply CHERRIES items to describe how the questionnaires were designed/deployed

If outcomes were obtained through online questionnaires, describe if they were validated for online use and apply CHERRIES items to describe how the questionnaires were designed/deployed [9].

	1	2	3	4	5	
subitem not at all important	◉	○	○	○	○	essential

Does your paper address subitem 6a-i?

Copy and paste relevant sections from manuscript text

Not applicable.

6a-ii) Describe whether and how “use” (including intensity of use/dosage) was defined/measured/monitored

Describe whether and how “use” (including intensity of use/dosage) was defined/measured/monitored (logins, logfile analysis, etc.). Use/adoption metrics are important process outcomes that should be reported in any ehealth trial.

	1	2	3	4	5	
subitem not at all important	○	○	○	○	○	essential

Does your paper address subitem 6a-ii?

Copy and paste relevant sections from manuscript text

"Forty-eight of the 57 (84%) participants completed at least one day of the eDiary protocol."

6a-iii) Describe whether, how, and when qualitative feedback from participants was obtained

Describe whether, how, and when qualitative feedback from participants was obtained (e.g., through emails, feedback forms, interviews, focus groups).

	1	2	3	4	5	
subitem not at all important	◉	○	○	○	○	essential

Does your paper address subitem 6a-iii?

Copy and paste relevant sections from manuscript text

We did not formally collect qualitative feedback from participants.

6b) Any changes to trial outcomes after the trial commenced, with reasons

Does your paper address CONSORT subitem 6b? *

Copy and paste relevant sections from the manuscript (include quotes in quotation marks "like this" to indicate direct quotes from your manuscript), or elaborate on this item by providing additional information not in the ms, or briefly explain why the item is not applicable/relevant for your study

Outcomes analysed in the present manuscript were not pre-specified at the start of the randomized controlled trial. This is clearly stated in the manuscript.

"The sample size calculation of our clinical trial was based on different outcomes than those reported herein. The eDiary outcomes that were analyzed are secondary outcomes of the clinical trial, which were conceptualized and implemented after the clinical trial started. Therefore, our study may not have had sufficient power to find differences in these secondary outcomes, resulting in potential false negatives when compared to primary outcomes analyses."

7a) How sample size was determined

NPT: When applicable, details of whether and how the clustering by care provides or centers was addressed

7a-i) Describe whether and how expected attrition was taken into account when calculating the sample size

Describe whether and how expected attrition was taken into account when calculating the sample size.

	1	2	3	4	5	
subitem not at all important	○	○	○	○	◉	essential

Does your paper address subitem 7a-i?

Copy and paste relevant sections from manuscript title (include quotes in quotation marks "like this" to indicate direct quotes from your manuscript), or elaborate on this item by providing additional information not in the ms, or briefly explain why the item is not applicable/relevant for your study

"The sample size was calculated based on the difference in the electroencephalogram alpha wave frequency between the groups (30%) with a probability of type I error of 5% and statistical power of 80%."

Attrition was not considered in the sample size calculation.

7b) When applicable, explanation of any interim analyses and stopping guidelines

Does your paper address CONSORT subitem 7b? *

Copy and paste relevant sections from the manuscript (include quotes in quotation marks "like this" to indicate direct quotes from your manuscript), or elaborate on this item by providing additional information not in the ms, or briefly explain why the item is not applicable/relevant for your study

No interim analyses were conducted during the study. Since the intervention was a home visiting program focused on promoting maternal and child health based on evidence based guidelines, no stopping guidelines were needed.

8a) Method used to generate the random allocation sequence

NPT: When applicable, how care providers were allocated to each trial group

Does your paper address CONSORT subitem 8a? *

Copy and paste relevant sections from the manuscript (include quotes in quotation marks "like this" to indicate direct quotes from your manuscript), or elaborate on this item by providing additional information not in the ms, or briefly explain why the item is not applicable/relevant for your study

www.random.org

8b) Type of randomisation; details of any restriction (such as blocking and block size)

Does your paper address CONSORT subitem 8b? *

Copy and paste relevant sections from the manuscript (include quotes in quotation marks "like this" to indicate direct quotes from your manuscript), or elaborate on this item by providing additional information not in the ms, or briefly explain why the item is not applicable/relevant for your study

"To avoid unbalanced groups, randomization was stratified according to the primary health care unit type and grandmother years of schooling."

9) Mechanism used to implement the random allocation sequence (such as sequentially numbered containers), describing any steps taken to conceal the sequence until interventions were assigned

Does your paper address CONSORT subitem 9? *

Copy and paste relevant sections from the manuscript (include quotes in quotation marks "like this" to indicate direct quotes from your manuscript), or elaborate on this item by providing additional information not in the ms, or briefly explain why the item is not applicable/relevant for your study

Allocation was conducted via Research Electronic Data Capture (REDCap).

10) Who generated the random allocation sequence, who enrolled participants, and who assigned participants to interventions

Does your paper address CONSORT subitem 10? *

Copy and paste relevant sections from the manuscript (include quotes in quotation marks "like this" to indicate direct quotes from your manuscript), or elaborate on this item by providing additional information not in the ms, or briefly explain why the item is not applicable/relevant for your study

Clinical trial management team conducted randomization, enrollment, and allocation.

11a) If done, who was blinded after assignment to interventions (for example, participants, care providers, those assessing outcomes) and how

NPT: Whether or not administering co-interventions were blinded to group assignment

11a-i) Specify who was blinded, and who wasn’t

Specify who was blinded, and who wasn’t. Usually, in web-based trials it is not possible to blind the participants [1, 3] (this should be clearly acknowledged), but it may be possible to blind outcome assessors, those doing data analysis or those administering co-interventions (if any).

	1	2	3	4	5	
subitem not at all important	○	○	○	○	○	essential

Does your paper address subitem 11a-i? *

Copy and paste relevant sections from the manuscript (include quotes in quotation marks "like this" to indicate direct quotes from your manuscript), or elaborate on this item by providing additional information not in the ms, or briefly explain why the item is not applicable/relevant for your study

Assessment team did not know randomization status of participants: "Participants were assessed by interviewers blinded to group allocation at 8-16 weeks of pregnancy (baseline), 30 weeks of pregnancy, and when the child was 3, 6, 12, 18, and 24 months of age."

Participants knew what intervention they were part of.

11a-ii) Discuss e.g., whether participants knew which intervention was the “intervention of interest” and which one was the “comparator”

Informed consent procedures (4a-ii) can create biases and certain expectations - discuss e.g., whether participants knew which intervention was the “intervention of interest” and which one was the “comparator”.

	1	2	3	4	5	
subitem not at all important	○	○	○	◉	○	essential

Does your paper address subitem 11a-ii?

Copy and paste relevant sections from the manuscript (include quotes in quotation marks "like this" to indicate direct quotes from your manuscript), or elaborate on this item by providing additional information not in the ms, or briefly explain why the item is not applicable/relevant for your study

Participants knew what intervention they were part of.

11b) If relevant, description of the similarity of interventions

(this item is usually not relevant for ehealth trials as it refers to similarity of a placebo or sham intervention to a active medication/intervention)

Does your paper address CONSORT subitem 11b? *

Copy and paste relevant sections from the manuscript (include quotes in quotation marks "like this" to indicate direct quotes from your manuscript), or elaborate on this item by providing additional information not in the ms, or briefly explain why the item is not applicable/relevant for your study

Intervention:

"Primeiros Laços is a home visiting intervention delivered by trained nurses that is tailored to first-time adolescent pregnant women and their children, which starts during the first 16 weeks of pregnancy until the child reaches 24 months of age. The frequency of visits was (a) biweekly during gestation and from 2 to 20 months of the child's age; (b) weekly during the first and last month of pregnancy, and puerperium; and (c) monthly from 21 to 24 months of the child's age. The Primeiros Laços program is based on three theoretical frameworks: attachment theory [28], self-efficacy theory [29], and the bioecological model [30]. This intervention adopts a learning-based approach and aims to have the child at the center of the mother's life, fostering her capacity to perceive and react to the child's needs, with the objective of improving maternal sensitivity to the child's behaviors and emotions. Primeiros Laços was developed by our team based on the Brazilian program Janelas de Oportunidades [31,32], Minding the Baby program [33], and Nurse-Family Partnership [34]. The development of the program was also informed with input from key national stakeholders involved in early childhood and maternal health research and advocacy."

Control:

"Participants allocated to the control group received care from Unified Health System (Sistema Único de Saúde), Brazil's public health system [37], according to national guidelines of the Ministry of Health [38-40] in line with World Health Organization guidelines. Prenatal and postnatal care is delivered by health units of the primary care system free of charge, focusing on preventive interventions, early detection of gestational risk, and referral to specialized health services in cases of high-risk pregnancies. [illegible]

12a) Statistical methods used to compare groups for primary and secondary outcomes

NPT: When applicable, details of whether and how the clustering by care providers or centers was addressed

Does your paper address CONSORT subitem 12a? *

Copy and paste relevant sections from the manuscript (include quotes in quotation marks "like this" to indicate direct quotes from your manuscript), or elaborate on this item by providing additional information not in the ms, or briefly explain why the item is not applicable/relevant for your study

"To examine intervention effects on eDiary outcomes, we used generalized estimating equation models [48]. Continuous outcomes were normalized using z-scores. Generalized estimating equation models are used to examine mean differences in normalized continuous outcomes and differences in predictive probabilities for categorical outcomes, both over multiple time points. The quasi-likelihood under the independence model criterion was used to indicate adequate correlation structures for longitudinal data that show a better fit to each model [49]. Fitted models were used to estimate and plot marginal mean scores for continuous outcomes and predictive probabilities for categorical outcomes at each time point. Time was entered as a continuous covariate in all models. Time trends were verified, and all models presented a linear trend [50]. All randomized participants that were contacted when their child was 18 months of age were included in the analysis, except for participants with missing data at all time points (n=9). Statistical tests were all two-sided and P values <.05 were considered statistically significant. Parameter estimates are reported with the 95% CI. Analyses were conducted using STATA 15.1 software."

12a-i) Imputation techniques to deal with attrition / missing values

Imputation techniques to deal with attrition / missing values: Not all participants will use the intervention/comparator as intended and attrition is typically high in ehealth trials. Specify how participants who did not use the application or dropped out from the trial were treated in the statistical analysis (a complete case analysis is strongly discouraged, and simple imputation techniques such as LOCF may also be problematic [4]).

	1	2	3	4	5	
subitem not at all important	○	○	○	○	○	essential

Does your paper address subitem 12a-i? *

Copy and paste relevant sections from the manuscript (include quotes in quotation marks "like this" to indicate direct quotes from your manuscript), or elaborate on this item by providing additional information not in the ms, or briefly explain why the item is not applicable/relevant for your study

Generalized estimating equation models can deal with missing data without the need for specific imputation techniques.

12b) Methods for additional analyses, such as subgroup analyses and adjusted analyses

Does your paper address CONSORT subitem 12b? *

Copy and paste relevant sections from the manuscript (include quotes in quotation marks "like this" to indicate direct quotes from your manuscript), or elaborate on this item by providing additional information not in the ms, or briefly explain why the item is not applicable/relevant for your study

Not applicable.

X26) REB/IRB Approval and Ethical Considerations [recommended as subheading under "Methods"] (not a CONSORT item)

X26-i) Comment on ethics committee approval

	1	2	3	4	5	
subitem not at all important	○	○	○	○	○	essential

Does your paper address subitem X26-i?

Copy and paste relevant sections from the manuscript (include quotes in quotation marks "like this" to indicate direct quotes from your manuscript), or elaborate on this item by providing additional information not in the ms, or briefly explain why the item is not applicable/relevant for your study

"Our study was approved by the Ethics Committee of the University of Sao Paulo Medical School, University Hospital of the University of Sao Paulo, and Sao Paulo Municipal Health Department. All participants and their primary caregiver signed written informed consent forms. The study was registered at clinicaltrials.gov (NCT02807818)."

x26-ii) Outline informed consent procedures

Outline informed consent procedures e.g., if consent was obtained offline or online (how? Checkbox, etc.?), and what information was provided (see 4a-ii). See [6] for some items to be included in informed consent documents.

	1	2	3	4	5	
subitem not at all important	○	○	○	○	○	essential

Does your paper address subitem X26-ii?

Copy and paste relevant sections from the manuscript (include quotes in quotation marks "like this" to indicate direct quotes from your manuscript), or elaborate on this item by providing additional information not in the ms, or briefly explain why the item is not applicable/relevant for your study

Consent was obtained offline in a face-to-face interview.

X26-iii) Safety and security procedures

Safety and security procedures, incl. privacy considerations, and any steps taken to reduce the likelihood or detection of harm (e.g., education and training, availability of a hotline)

	1	2	3	4	5	
subitem not at all important	○	○	○	○	○	essential

Does your paper address subitem X26-iii?

Copy and paste relevant sections from the manuscript (include quotes in quotation marks "like this" to indicate direct quotes from your manuscript), or elaborate on this item by providing additional information not in the ms, or briefly explain why the item is not applicable/relevant for your study

All home visitors and assessors were well trained experienced health professionals that were available when participants had questions, problems, or issues. Home visitors and assessors were supervised weekly to ensure participants were receiving the best possible care and attention.

RESULTS

13a) For each group, the numbers of participants who were randomly assigned, received intended treatment, and were analysed for the primary outcome

NPT: The number of care providers or centers performing the intervention in each group and the number of patients treated by each care provider in each center

Does your paper address CONSORT subitem 13a? *

Copy and paste relevant sections from the manuscript (include quotes in quotation marks "like this" to indicate direct quotes from your manuscript), or elaborate on this item by providing additional information not in the ms, or briefly explain why the item is not applicable/relevant for your study

"A total of 169 pregnant youth were assessed for eligibility criteria, 80 of whom were included and randomized to the intervention (n=40) and control (care as usual) group (n=40)."

"We were able to contact 57/80 (71%) participants (29 in the intervention group and 28 in the control group) when the child was 18 months old."

13b) For each group, losses and exclusions after randomisation, together with reasons

Does your paper address CONSORT subitem 13b? (NOTE: Preferably, this is shown in a CONSORT flow diagram) *

Copy and paste relevant sections from the manuscript (include quotes in quotation marks "like this" to indicate direct quotes from your manuscript), or elaborate on this item by providing additional information not in the ms, or briefly explain why the item is not applicable/relevant for your study

See CONSORT diagram.

13b-i) Attrition diagram

Strongly recommended: An attrition diagram (e.g., proportion of participants still logging in or using the intervention/comparator in each group plotted over time, similar to a survival curve) or other figures or tables demonstrating usage/dose/engagement.

	1	2	3	4	5	
subitem not at all important	○	○	○	●	○	essential

Does your paper address subitem 13b-i?

Copy and paste relevant sections from the manuscript or cite the figure number if applicable (include quotes in quotation marks "like this" to indicate direct quotes from your manuscript), or elaborate on this item by providing additional information not in the ms, or briefly explain why the item is not applicable/relevant for your study

"Figure 1 shows the Consolidated Standards of Reporting Trials (CONSORT) flow chart for participant selection and grouping throughout the trial."

14a) Dates defining the periods of recruitment and follow-up

Does your paper address CONSORT subitem 14a? *

Copy and paste relevant sections from the manuscript (include quotes in quotation marks "like this" to indicate direct quotes from your manuscript), or elaborate on this item by providing additional information not in the ms, or briefly explain why the item is not applicable/relevant for your study

Recruitment: "From June to September 2015, a total of 169 pregnant youth were assessed for eligibility criteria".

Follow-up period: "From June to October 2017, trained psychologists visited participants to provide support on downloading, installing, and using the app."

14a-i) Indicate if critical “secular events” fell into the study period

Indicate if critical “secular events” fell into the study period, e.g., significant changes in Internet resources available or “changes in computer hardware or Internet delivery resources”

	1	2	3	4	5	
subitem not at all important	(●)	( )	( )	( )	( )	essential

Does your paper address subitem 14a-i?

Copy and paste relevant sections from the manuscript (include quotes in quotation marks "like this" to indicate direct quotes from your manuscript), or elaborate on this item by providing additional information not in the ms, or briefly explain why the item is not applicable/relevant for your study

Not applicable.

14b) Why the trial ended or was stopped (early)

Does your paper address CONSORT subitem 14b? *

Copy and paste relevant sections from the manuscript (include quotes in quotation marks "like this" to indicate direct quotes from your manuscript), or elaborate on this item by providing additional information not in the ms, or briefly explain why the item is not applicable/relevant for your study

The trial was not stopped earlier than planned.

15) A table showing baseline demographic and clinical characteristics for each group

NPT: When applicable, a description of care providers (case volume, qualification, expertise, etc.) and centers (volume) in each group

Does your paper address CONSORT subitem 15? *

Copy and paste relevant sections from the manuscript (include quotes in quotation marks "like this" to indicate direct quotes from your manuscript), or elaborate on this item by providing additional information not in the ms, or briefly explain why the item is not applicable/relevant for your study

"Table 1. Baseline sample characteristics (N=80)."

15-i) Report demographics associated with digital divide issues

In ehealth trials it is particularly important to report demographics associated with digital divide issues, such as age, education, gender, social-economic status, computer/Internet/ehealth literacy of the participants, if known.

	1	2	3	4	5	
subitem not at all important	○	○	○	○	○	essential

Does your paper address subitem 15-i? *

Copy and paste relevant sections from the manuscript (include quotes in quotation marks "like this" to indicate direct quotes from your manuscript), or elaborate on this item by providing additional information not in the ms, or briefly explain why the item is not applicable/relevant for your study

Characteristics such as age, education, gender, social-economic status are reported in table 1.

16) For each group, number of participants (denominator) included in each analysis and whether the analysis was by original assigned groups

16-i) Report multiple “denominators” and provide definitions

Report multiple “denominators” and provide definitions: Report N’s (and effect sizes) “across a range of study participation [and use] thresholds” [1], e.g., N exposed, N consented, N used more than x times, N used more than y weeks, N participants “used” the intervention/comparator at specific pre-defined time points of interest (in absolute and relative numbers per group). Always clearly define “use” of the intervention.

	1	2	3	4	5	
subitem not at all important	○	○	○	○	○	essential

Does your paper address subitem 16-i? *

Copy and paste relevant sections from the manuscript (include quotes in quotation marks "like this" to indicate direct quotes from your manuscript), or elaborate on this item by providing additional information not in the ms, or briefly explain why the item is not applicable/relevant for your study

"Forty-eight of the 57 (84%) participants completed at least one day of the eDiary protocol. The daily compliance rate ranged from 49% to 70% (Figure 2)."

16-ii) Primary analysis should be intent-to-treat

Primary analysis should be intent-to-treat, secondary analyses could include comparing only “users”, with the appropriate caveats that this is no longer a randomized sample (see 18-i).

	1	2	3	4	5	
subitem not at all important	○	○	○	○	◉	essential

Does your paper address subitem 16-ii?

Copy and paste relevant sections from the manuscript (include quotes in quotation marks "like this" to indicate direct quotes from your manuscript), or elaborate on this item by providing additional information not in the ms, or briefly explain why the item is not applicable/relevant for your study

Analysis were intent-to-treat.

17a) For each primary and secondary outcome, results for each group, and the estimated effect size and its precision (such as 95% confidence interval)

Does your paper address CONSORT subitem 17a? *

Copy and paste relevant sections from the manuscript (include quotes in quotation marks "like this" to indicate direct quotes from your manuscript), or elaborate on this item by providing additional information not in the ms, or briefly explain why the item is not applicable/relevant for your study

"Figure 3 and Figure 4 depict the results of the generalized estimating equations models with trajectories of outcomes. Overall, the results showed a significant effect of the intervention on parental well-being (B=0.32, 95% CI [0.06, 0.58], P=.01) and increased behavior of the mother telling a story or singing to the child (odds ratio=2.33, 95% CI [1.20, 4.50], P=.01) (Figure 4)."

17a-i) Presentation of process outcomes such as metrics of use and intensity of use

In addition to primary/secondary (clinical) outcomes, the presentation of process outcomes such as metrics of use and intensity of use (dose, exposure) and their operational definitions is critical. This does not only refer to metrics of attrition (13-b) (often a binary variable), but also to more continuous exposure metrics such as "average session length". These must be accompanied by a technical description how a metric like a "session" is defined (e.g., timeout after idle time) [1] (report under item 6a).

	1	2	3	4	5	
subitem not at all important	◉	○	○	○	○	essential

Does your paper address subitem 17a-i?

Copy and paste relevant sections from the manuscript (include quotes in quotation marks "like this" to indicate direct quotes from your manuscript), or elaborate on this item by providing additional information not in the ms, or briefly explain why the item is not applicable/relevant for your study

Not applicable.

17b) For binary outcomes, presentation of both absolute and relative effect sizes is recommended

Does your paper address CONSORT subitem 17b? *

Copy and paste relevant sections from the manuscript (include quotes in quotation marks "like this" to indicate direct quotes from your manuscript), or elaborate on this item by providing additional information not in the ms, or briefly explain why the item is not applicable/relevant for your study

For absolute effect: "Table 3. Frequency of categorical outcomes per day (sample size of categories varies due to participant non-response)."

18) Results of any other analyses performed, including subgroup analyses and adjusted analyses, distinguishing pre-specified from exploratory

Does your paper address CONSORT subitem 18? *

Copy and paste relevant sections from the manuscript (include quotes in quotation marks "like this" to indicate direct quotes from your manuscript), or elaborate on this item by providing additional information not in the ms, or briefly explain why the item is not applicable/relevant for your study

Not applicable.

18-i) Subgroup analysis of comparing only users

A subgroup analysis of comparing only users is not uncommon in ehealth trials, but if done, it must be stressed that this is a self-selected sample and no longer an unbiased sample from a randomized trial (see 16-iii).

	1	2	3	4	5	
subitem not at all important	(●)	( )	( )	( )	( )	essential

Does your paper address subitem 18-i?

Copy and paste relevant sections from the manuscript (include quotes in quotation marks "like this" to indicate direct quotes from your manuscript), or elaborate on this item by providing additional information not in the ms, or briefly explain why the item is not applicable/relevant for your study

Not applicable.

19) All important harms or unintended effects in each group

(for specific guidance see CONSORT for harms)

Does your paper address CONSORT subitem 19? *

Copy and paste relevant sections from the manuscript (include quotes in quotation marks "like this" to indicate direct quotes from your manuscript), or elaborate on this item by providing additional information not in the ms, or briefly explain why the item is not applicable/relevant for your study

No harm or unintended effects were observed throughout the trial.

19-i) Include privacy breaches, technical problems

Include privacy breaches, technical problems. This does not only include physical “harm” to participants, but also incidents such as perceived or real privacy breaches [1], technical problems, and other unexpected/unintended incidents. “Unintended effects” also includes unintended positive effects [2].

	1	2	3	4	5	
subitem not at all important	○	○	○	○	○	essential

Does your paper address subitem 19-i?

Copy and paste relevant sections from the manuscript (include quotes in quotation marks "like this" to indicate direct quotes from your manuscript), or elaborate on this item by providing additional information not in the ms, or briefly explain why the item is not applicable/relevant for your study

No privacy breaches or technical problems happened during the study.

19-ii) Include qualitative feedback from participants or observations from staff/researchers

Include qualitative feedback from participants or observations from staff/researchers, if available, on strengths and shortcomings of the application, especially if they point to unintended/unexpected effects or uses. This includes (if available) reasons for why people did or did not use the application as intended by the developers.

	1	2	3	4	5	
subitem not at all important	○	○	○	○	○	essential

Does your paper address subitem 19-ii?

Copy and paste relevant sections from the manuscript (include quotes in quotation marks "like this" to indicate direct quotes from your manuscript), or elaborate on this item by providing additional information not in the ms, or briefly explain why the item is not applicable/relevant for your study

We did not formally collect qualitative feedback from participants or observations from staff/researchers.

DISCUSSION

22) Interpretation consistent with results, balancing benefits and harms, and considering other relevant evidence

NPT: In addition, take into account the choice of the comparator, lack of or partial blinding, and unequal expertise of care providers or centers in each group

22-i) Restate study questions and summarize the answers suggested by the data, starting with primary outcomes and process outcomes (use)

Restate study questions and summarize the answers suggested by the data, starting with primary outcomes and process outcomes (use).

	1	2	3	4	5	
subitem not at all important	○	○	○	○	○	essential

Does your paper address subitem 22-i? *

Copy and paste relevant sections from the manuscript (include quotes in quotation marks "like this" to indicate direct quotes from your manuscript), or elaborate on this item by providing additional information not in the ms, or briefly explain why the item is not applicable/relevant for your study

"This is the first ECD randomized controlled trial testing an intervention using a smartphone-based ambulatory assessment to measure outcomes. The results showed that our nurse home visiting program had a positive effect on parental well-being and maternal parenting as measured by a smartphone eDiary."

22-ii) Highlight unanswered new questions, suggest future research

Highlight unanswered new questions, suggest future research.

	1	2	3	4	5	
subitem not at all important	○	○	○	○	○	essential

Does your paper address subitem 22-ii?

Copy and paste relevant sections from the manuscript (include quotes in quotation marks "like this" to indicate direct quotes from your manuscript), or elaborate on this item by providing additional information not in the ms, or briefly explain why the item is not applicable/relevant for your study

"Moreover, future implementations of smartphone-based assessments in low-income areas should consider that some people may not own a smartphone. This approach also may not be suited for rural areas, especially in LMICs, where poverty is more predominant."

"Second, our findings demonstrate the potential for future ECD intervention studies to implement ambulatory assessment in LMICs via smartphones for measuring mother and child behaviors. More frequent assessments of maternal parenting behaviors and well-being should be implemented to further enhance temporal and ecological validity, and to also expand the scope of measured behaviors."

20) Trial limitations, addressing sources of potential bias, imprecision, and, if relevant, multiplicity of analyses

20-i) Typical limitations in ehealth trials

Typical limitations in ehealth trials: Participants in ehealth trials are rarely blinded. Ehealth trials often look at a multiplicity of outcomes, increasing risk for a Type I error. Discuss biases due to non-use of the intervention/usability issues, biases through informed consent procedures, unexpected events.

	1	2	3	4	5	
subitem not at all important	○	○	○	○	●	essential

Does your paper address subitem 20-i? *

Copy and paste relevant sections from the manuscript (include quotes in quotation marks "like this" to indicate direct quotes from your manuscript), or elaborate on this item by providing additional information not in the ms, or briefly explain why the item is not applicable/relevant for your study

"Our findings should be viewed in light of some limitations. The sample size calculation of our clinical trial was based on different outcomes than those reported herein. The eDiary outcomes that were analyzed are secondary outcomes of the clinical trial, which were conceptualized and implemented after the clinical trial started. Therefore, our study may not have had sufficient power to find differences in these secondary outcomes, resulting in potential false negatives when compared to primary outcomes analyses. Additionally, our sample size limited the scope of our analyses. For instance, it would be interesting to use a structural equation modeling approach such as growth curves to validate the constructs assessed; however, the small sample sizes did not allow for convergence of these models. Moreover, the lack of variability and a ceiling effect in some variables may have also influenced these potential analyses. Therefore, even though we presented findings suggesting that we adequately measured the constructs of interest, we were not able to validate these measures quantitatively. However, we found that maternal parenting behaviors were negatively associated with maternal depression measured at the same time point [25], which is an expected finding since it is known that depressive symptoms can influence parenting. In addition, our assessment of maternal parenting was based exclusively on the frequency of behaviors, and we did not collect data on the quality of the interaction between the participants and their children. The quality of maternal parenting is known to be crucial for child development and the mother-child relationship. In addition, the use of eDiary self-report assessment may have influenced the findings due to effects of social desirability bias [63] or the participants' awareness of being part of an ECD study [64]. However, this is unlikely since we detected low rates of some maternal parenting behaviors that would be considered socially approved. Moreover, we included a subgroup of participants who did not own a smartphone at the time of trial commencement. This was surprising since 86% of participants reported owning a smartphone at baseline. We found that it is a common practice among this population to frequently exchange, borrow, or buy smartphones. Furthermore, some participants owned old smartphones with limited hardware in terms of performance as well as with old operating systems, preventing them from properly using the smartphone app. We were able to include these participants by lending them a smartphone, which could have influenced our results. However, we did not find differences in outcomes between participants with their own smartphone compared with those who borrowed smartphones."

21) Generalisability (external validity, applicability) of the trial findings

NPT: External validity of the trial findings according to the intervention, comparators, patients, and care providers or centers involved in the trial

21-i) Generalizability to other populations

Generalizability to other populations: In particular, discuss generalizability to a general Internet population, outside of a RCT setting, and general patient population, including applicability of the study results for other organizations

	1	2	3	4	5	
subitem not at all important	○	○	◉	○	○	essential

Does your paper address subitem 21-i?

Copy and paste relevant sections from the manuscript (include quotes in quotation marks "like this" to indicate direct quotes from your manuscript), or elaborate on this item by providing additional information not in the ms, or briefly explain why the item is not applicable/relevant for your study

Our results are based on a very specific sample derived from a population of pregnant youth living in adverse conditions in the the western region of Sao Paulo. Therefore, it is difficult to generalize our findings. Further studies are warranted.

21-ii) Discuss if there were elements in the RCT that would be different in a routine application setting

Discuss if there were elements in the RCT that would be different in a routine application setting (e.g., prompts/reminders, more human involvement, training sessions or other co-interventions) and what impact the omission of these elements could have on use, adoption, or outcomes if the intervention is applied outside of a RCT setting.

	1	2	3	4	5	
subitem not at all important	○	○	○	○	○	essential

Does your paper address subitem 21-ii?

Copy and paste relevant sections from the manuscript (include quotes in quotation marks "like this" to indicate direct quotes from your manuscript), or elaborate on this item by providing additional information not in the ms, or briefly explain why the item is not applicable/relevant for your study

We did not assess elements in the RCT that would be different in a routine application setting.

OTHER INFORMATION

23) Registration number and name of trial registry

Does your paper address CONSORT subitem 23? *

Copy and paste relevant sections from the manuscript (include quotes in quotation marks "like this" to indicate direct quotes from your manuscript), or elaborate on this item by providing additional information not in the ms, or briefly explain why the item is not applicable/relevant for your study

"The study was registered at clinicaltrials.gov (NCT02807818)."

24) Where the full trial protocol can be accessed, if available

Does your paper address CONSORT subitem 24? *

Cite a Multimedia Appendix, other reference, or copy and paste relevant sections from the manuscript (include quotes in quotation marks "like this" to indicate direct quotes from your manuscript), or elaborate on this item by providing additional information not in the ms, or briefly explain why the item is not applicable/relevant for your study

Not available yet.

25) Sources of funding and other support (such as supply of drugs), role of funders

Does your paper address CONSORT subitem 25? *

Copy and paste relevant sections from the manuscript (include quotes in quotation marks "like this" to indicate direct quotes from your manuscript), or elaborate on this item by providing additional information not in the ms, or briefly explain why the item is not applicable/relevant for your study

"This work was supported by Grand Challenges Canada (GCC), Fundação Maria Cecília Souto Vidigal (0722-03), the Bill & Melinda Gates Foundation (OPP1142172), Fundação de Amparo à Pesquisa do Estado de São Paulo (FAPESP) (2016/13451-9), Conselho Nacional de Desenvolvimento Científico e Tecnológico (420267/2016-6), and Companhia Brasileira de Metalurgia e Mineração."

X27) Conflicts of Interest (not a CONSORT item)

X27-i) State the relation of the study team towards the system being evaluated

In addition to the usual declaration of interests (financial or otherwise), also state the relation of the study team towards the system being evaluated, i.e., state if the authors/evaluators are distinct from or identical with the developers/sponsors of the intervention.

	1	2	3	4	5	
subitem not at all important	○	○	○	○	○	essential

Does your paper address subitem X27-i?

Copy and paste relevant sections from the manuscript (include quotes in quotation marks "like this" to indicate direct quotes from your manuscript), or elaborate on this item by providing additional information not in the ms, or briefly explain why the item is not applicable/relevant for your study

No conflict of interest towards the system being evaluated to be declared.

About the CONSORT EHEALTH checklist

As a result of using this checklist, did you make changes in your manuscript? *

- ○ yes, major changes
- ◉ yes, minor changes
- ○ no

What were the most important changes you made as a result of using this checklist?

95% Confidence Intervals.

How much time did you spend on going through the checklist INCLUDING making changes in your manuscript *

2 hours.

As a result of using this checklist, do you think your manuscript has improved? *

- ◉ yes
- ○ no
- ○ Other:

Would you like to become involved in the CONSORT EHEALTH group?

This would involve for example becoming involved in participating in a workshop and writing an "Explanation and Elaboration" document

- ( ) yes
- (•) no
- ( ) Other:

Any other comments or questions on CONSORT EHEALTH

It must be noted that the intervention described in our manuscript is not electronic or digital. What we report in the manuscript is a outcome assessment conducted using a smartphone app in the context of a randomized controlled trial designed to test a nurse home visiting program for pregnant youth living under adverse conditions.

STOP - Save this form as PDF before you click submit

To generate a record that you filled in this form, we recommend to generate a PDF of this page (on a Mac, simply select "print" and then select "print as PDF") before you submit it.

When you submit your (revised) paper to JMIR, please upload the PDF as supplementary file.

Don't worry if some text in the textboxes is cut off, as we still have the complete information in our database. Thank you!

Final step: Click submit !

Click submit so we have your answers in our database!

Submit

Never submit passwords through Google Forms.

This content is neither created nor endorsed by Google. Report Abuse - Terms of Service - Privacy Policy

Google Forms
